# MicroRNA–mRNA Networks in Skeletal Muscle of Tailored Pig Models for Dystrophinopathies

**DOI:** 10.1002/jcsm.70337

**Published:** 2026-09-07

**Authors:** Sarah Reschke, Alexander Graf, Frieder Hadlich, Florian Jaudas, Thomas Fröhlich, Michael Stirm, Nikolai Klymiuk, Stefan Krebs, Eckhard Wolf, Asghar Ali

**Affiliations:** ^1^ Chair of Molecular Animal Breeding and Biotechnology, Gene Center and Department of Veterinary Sciences LMU Munich Munich Germany; ^2^ Laboratory for Functional Genome Analysis (LAFUGA), Gene Center LMU Munich Munich Germany; ^3^ Working Group Physiological Genomics Research Institute for Farm Animal Biology (FBN) Dummerstorf Germany; ^4^ Center for Innovative Medical Models (CiMM) LMU Munich Oberschleißheim Germany; ^5^ Klinik und Poliklinik für Innere Medizin I TUM Klinikum rechts der Isar Munich Germany; ^6^ Interfaculty Center for Endocrine and Cardiovascular Disease Network Modelling and Clinical Transfer (ICONLMU) LMU Munich Munich Germany; ^7^ German Center for Child and Adolescent Diseases (DZKJ) Munich Germany

**Keywords:** Becker muscular dystrophy (BMD), biomarkers, Duchenne muscular dystrophy (DMD), enrichment analysis, exon skipping

## Abstract

**Background:**

Duchenne muscular dystrophy (DMD) and Becker muscular dystrophy (BMD) are X‐linked dystrophinopathies caused by mutations in the dystrophin (*DMD*) gene. A common DMD‐causing mutation in humans is exon 52 deletion (*DMD*Δ52), which disrupts the reading frame and abolishes dystrophin expression. Therapeutic skipping of exon 51 or 53 can restore the reading frame, producing a truncated but functional protein and generating a BMD‐like phenotype. Porcine models recapitulating *DMD*Δ52 (DMD) and *DMD*Δ51–52 (BMD‐like) were used to identify molecular differences and condition‐specific miRNA–mRNA networks.

**Methods:**

Skeletal muscle (triceps brachii) from four DMD, four BMD, and five wild‐type (WT) pigs at 3.5 months of age underwent stranded total RNA‐seq and small RNA‐seq. Differentially expressed mRNAs (|log2FC| ≥ 1, adj. *p* ≤ 0.05) and miRNAs (adj. *p* ≤ 0.05) were identified with DESeq2. miRNA–mRNA networks were constructed using RNAhybrid predictions (MFE < −25 kcal/mol, seed pairing) filtered by inverse Pearson correlation.

**Results:**

Compared with WT, DMD muscle exhibited 1440 upregulated and 487 downregulated genes, characterized by strong repression of structural, contractile, calcium‐handling and metabolic genes (e.g., *MYBPC2*, *MYL3*, *MYLK2*, *CACNA2D3*, *CACNA2D4*) and marked upregulation of inflammatory mediators and innate immune receptors (e.g., *IL6*, *IL18*, *IL1R1*, *CCR1/2/5*, *TLR1/2/4/7/9*). In contrast, BMD muscle showed partial restoration of these pathways and clustered closer to WT in global expression profiles. Distinct miRNA signatures were observed between DMD and BMD. Differential expression analysis identified 22 upregulated and 12 downregulated miRNAs in DMD versus WT and 36 upregulated and 21 downregulated miRNAs in BMD versus WT. Integration of miRNA and mRNA data yielded extensive regulatory networks (1013 unique pairs for upregulated miRNAs in DMD; 2679 pairs for downregulated miRNAs in BMD). Two condition‐specific miRNAs emerged as strong biomarker candidates: ssc‐miR‐296‐3p (upregulated exclusively in DMD, targeting 228 genes enriched in muscle structure and fatty acid metabolism) and ssc‐miR‐423‐5p (elevated specifically in BMD, targeting 67 genes involved in calcium signalling and tissue development). Several dysregulated miRNAs, including miR‐199a‐5p and miR‐199b, overlapped with those reported in human DMD and other muscular dystrophies.

**Conclusions:**

Exon 51 skipping in the *DMD*Δ52 background partially restores key transcriptional programmes in skeletal muscle but does not fully normalize them to WT patterns. The identification of condition‐specific miRNAs highlights post‐transcriptional regulatory differences between DMD and BMD, positioning them as promising biomarkers and therapeutic targets. These findings underscore the translational value of porcine dystrophinopathy models for mechanistic studies and preclinical evaluation of RNA‐targeted interventions.

## 1 Introduction

Duchenne muscular dystrophy (DMD) and Becker muscular dystrophy (BMD) are among the most common muscular dystrophies, affecting approximately 4.8 and 1.5 individuals per 100 000 in the general population, respectively [1]. Both are X‐linked disorders caused by mutations in the dystrophin (*DMD*) gene, one of the largest known protein‐coding genes in mammals [2]. DMD typically results from single‐ or multi‐exon deletions that often cluster between exons 45 and 55 [3], disrupting the reading frame, abolishing dystrophin expression and leading to progressive muscle degeneration and premature death from cardiorespiratory failure [4, 5]. In contrast, in BMD, in‐frame mutations allow the synthesis of partially functional dystrophin, resulting in a milder clinical phenotype [6]. A well‐characterized pathogenic *DMD* mutation is the deletion of exon 52 (*DMD*Δ52), which causes a frameshift loss of dystrophin protein [7]. This mutation is particularly amenable to exon‐skipping therapies targeting exon 51 or 53 to restore the reading frame and generate a shortened yet functional protein [8].

Porcine models provide a physiologically relevant platform for studying dystrophinopathies due to their close resemblance to human muscle structure and biology [9]. Using somatic cell nuclear transfer (SCNT), we generated DMD pigs lacking exon 52 (*DMD*Δ52), which displayed dystrophin deficiency, impaired mobility, and progressive muscle pathology, with a lifespan of 3–9 months [10, 11]. BMD pigs were subsequently generated by deleting exon 51 in *DMD*Δ52 cells, thereby restoring the reading frame (*DMD*Δ51–52). These animals expressed dystrophin in skeletal muscle and myocardium and showed markedly improved pathology, providing a robust model for exon‐skipping–mediated therapeutic correction [12]. Functional analysis showed that *DMD*Δ52 pigs (DMD model) exhibited severely reduced muscle force, whereas *DMD*Δ51–52 pigs (BMD model) displayed partial restoration but still below wild‐type (WT) levels [13]. Twitch and tetanic contractions remained impaired in both models, though BMD pigs showed improved performance compared to DMD [13].

A key application of large‐animal dystrophinopathy models is the discovery of molecular biomarkers and therapeutic targets. In this context, circulating microRNAs (miRNAs), non‐coding RNAs regulating post‐transcriptional gene expression, have emerged as minimally invasive indicators of systemic alterations in biological processes. For instance, muscle‐enriched miRNAs such as miR‐1, miR‐133, and miR‐206 are consistently dysregulated in dystrophinopathies and readily detectable in blood [14, 15]. Previous studies have shown that miRNA dysregulation in skeletal muscle and circulation can serve as potential biomarkers for muscular dystrophies in humans [16, 17]. Emerging evidence further indicates that distinct miRNA expression signatures can differentiate DMD from BMD [16, 18]. Yet, miRNA–mRNA regulatory interactions remain poorly defined in large‐animal models of dystrophinopathies. To address this gap, we integrated skeletal muscle miRNA and mRNA expression profiles from our porcine models of DMD and BMD. Our findings demonstrate that therapeutic exon 51 deletion in the *DMD*Δ52 background partially restored transcriptional network alterations characteristic of DMD, revealing condition‐specific regulatory circuits and pinpointing miRNAs with strong diagnostic and therapeutic potential. Together, these results clarify key miRNA–mRNA interactions in dystrophinopathies and underscore the value of clinically relevant porcine models for mechanistic and translational research.

## 2 Materials and Methods

### 2.1 Animals

All animal experiments were performed in accordance with the German Animal Welfare Act and Directive 2010/63/EU and were approved by the responsible authority, the Government of Upper Bavaria (ROB‐55.2‐2532.Vet_02‐17‐136). The microbiological status of the herd was routinely monitored [free from notifiable animal diseases according to Section 4 of the German Animal Health Act (TierGesG) and the Ordinance on Notifiable Animal Diseases (TierSeuchAnzV)]. The generation of porcine dystrophinopathy models has been described previously. Specifically, the deletion of exon 52 in the *DMD* gene and the establishment of the corresponding DMD pig line were reported in [10, 11]. Likewise, the deletion of exon 51 from *DMD*Δ52 pigs and the establishment of the BMD pig line were described in [12, 19]. For the present study, skeletal muscle samples were obtained from the triceps brachii of 13 animals at a similar age of 3.5 months, comprising four DMD pigs, four BMD pigs and five WT controls. Biopsies were collected under standardized conditions to minimize variability, immediately frozen and later used for RNA isolation.

### 2.2 RNA Isolation

Total RNA was extracted from powdered skeletal muscle using TRIzol reagent (Thermo Fisher Scientific, Waltham, MA, USA) in combination with Phasemaker tubes (Thermo Fisher Scientific) following the manufacturer's guidelines. RNA quality was evaluated using an Agilent 2100 Bioanalyzer with the RNA Nano 6000 Kit (Agilent Technologies, Santa Clara, CA, USA), and RNA concentration was determined with a NanoDrop ND‐1000 spectrophotometer (Thermo Fisher Scientific).

### 2.3 Gene Expression Profiling

Isolated RNA of 1 μg was purified using Mag‐Bind Total Pure NGS magnetic beads (Machery & Nagel, Düren, Germany). Stranded total RNA‐seq libraries were prepared from 4.5 ng RNA using the SMARTer Stranded Total RNA‐Seq Kit v3—Pico Input Mammalian Kit (TaKaRa Bio, Fitchburg, WI, USA), and PCR products were purified with Mag‐Bind beads (Machery & Nagel, Düren, Germany). Libraries were sequenced on an Illumina NextSeq 1000 (Illumina, San Diego, CA, USA) with a read length of 60 bp in paired‐end read mode. Reads were demultiplexed and quality‐checked with FastQC (v.0.73). Adapter sequences and low‐quality bases were trimmed using Trim Galore! (v.0.6.7). UMI barcodes were extracted using UMI‐tools extract (v.1.1.2), and six nucleotides from the UMI linker were trimmed from read2 using fastp (v.0.23.2). Reads were aligned to the *Sus scrofa* 11.1 genome using STAR (v.2.7.10b) with RefSeq gene annotations, deduplicated with UMI‐tools, and gene counts generated with featureCounts. Differential expression analysis was performed using DESeq2 (v.2.11.40.7), with genes considered significantly differentially expressed at|log2FC| ≥ 1 and adjusted *p* ≤ 0.05.

### 2.4 MicroRNA Analysis

Small RNA libraries were prepared from 460 ng of total RNA using the SMARTer smRNA Seq Kit for Illumina (TaKaRa Bio, Fitchburg, WI, USA) and purified with Mag‐Bind Total Pure NGS (Machery & Nagel, Düren, Germany). Sequencing was performed on the Illumina NextSeq 1000 instrument (Illumina, San Diego, CA, USA) with a read length of 2 × 60 bp in paired‐end read mode. Although mature miRNAs are short, libraries were sequenced in paired‐end mode, as this is the standard configuration in our facility. Reads were demultiplexed and quality‐checked with FastQC (v.0.73). Paired reads were merged with SeqPrep (v.0.2.2), and adapters were trimmed with Cutadapt (v.4.4). Read collapsing and genome mapping done using miRDeep2 Mapper (v.2.0.0). MiRNAs were then quantified using the miRDeep2 Quantifier module, which utilizes precursor and mature sequences for *S. scrofa* from miRBase (v.22.1). Differential miRNA expression was assessed with DESeq2 (v.2.11.40.7) using adjusted *p* ≤ 0.05 as the significance cutoff. Volcano plots were generated using ggplot2 in R. Additionally, heatmaps were created from variance‐stabilized (VST) values, which were previously z‐score normalized, for both mRNA and microRNA data using the pheatmap package. Principal component analysis (PCA) plots based on the first two principal components were generated using the DESeq2 package for both mRNA and microRNA data.

### 2.5 Prediction of Downstream Targets of miRNAs

To predict downstream target genes of differentially expressed miRNAs, 3′UTR, 5′UTR and CDS sequences were retrieved from the *S. scrofa* genome (Sscrofa11.1; Ensembl v112), comprising 17 299 3′UTRs, 17 277 5′UTRs and 20 987 CDSs. Each sequence was split into overlapping 2000‐bp fragments with a 50‐bp overlap, allowing for comprehensive scanning. Full‐length mature miRNA sequences were used for target prediction with RNAhybrid (v.2.1.2), applying stringent criteria: one binding site per transcript, human‐based *p*‐value distribution, minimum free energy (MFE) < −25 kcal/mol and a helix constraint at positions 2–7 to enforce seed‐region pairing. Predicted miRNA–mRNA pairs with binding sites in 3′UTRs were retained for further analysis. The expression profiles of miRNAs and their corresponding mRNAs were integrated, and Pearson correlation coefficients were calculated for each miRNA–mRNA pair. Only significantly inverse correlations, consistent with the canonical repressive role of miRNAs, were selected for downstream analyses, yielding a high‐confidence set of biologically meaningful regulatory interactions.

### 2.6 Enrichment Analysis

An integrative analysis of differentially expressed miRNAs and mRNAs was conducted across three pairwise comparisons: DMD versus WT, BMD versus WT and BMD versus DMD. Venn diagrams were generated to identify shared and unique sets of significantly dysregulated transcripts, allowing classification of genes as either commonly altered in both dystrophic models or uniquely associated with DMD or BMD. Genes uniquely upregulated or downregulated in each condition were subjected to functional enrichment analysis. KEGG pathway and Gene Ontology (GO) enrichment analyses (biological process, cellular component and molecular function) were performed using the GO/Pathway Enrichment module of the SRplot web server [20]. An analogous enrichment analysis was subsequently applied to the set of genes uniquely downregulated in the DMD or BMD group.

To characterize regulatory interactions between differentially expressed miRNAs and their inversely correlated target genes, enrichment analyses were carried out in Cytoscape (v.3.10.2) using ClueGO (v.2.5.10) and CluePedia (v.1.5.10). As a focused analysis, first, miRNAs upregulated in BMD compared with DMD and their negatively correlated target genes were analysed for KEGG and GO enrichment. Subsequently, miRNAs were downregulated in BMD compared to DMD, and their negatively correlated target genes were subjected to enrichment analysis. ClueGO enrichment used the hypergeometric test with the Benjamini–Hochberg correction, with the *S. scrofa* genome as the background. Pathways and GO categories with *p* ≤ 0.05 were considered significantly enriched.

## 3 Results

### 3.1 Altered mRNA Profiles in DMD and BMD Muscle

**FIGURE 1:**
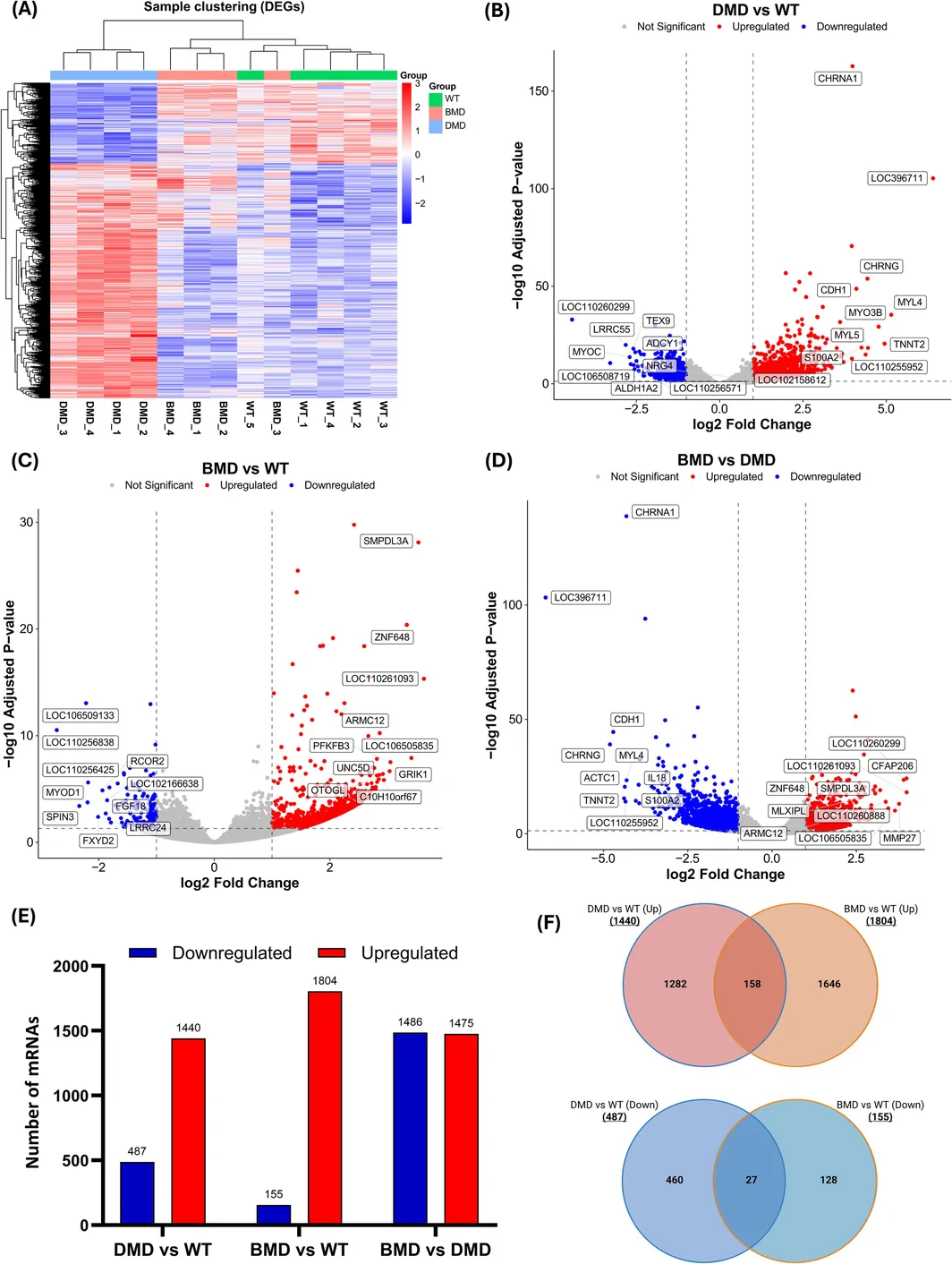
Differential gene expression analysis. (A) Heatmap showing the comparison of DEGs in all groups. Each column represents a biological replicate, and each row corresponds to a DEG (|log2FC| ≥ 1, adjusted *p* ≤ 0.05). (B‐D) Volcano plots of DEGs in pairwise comparisons. (E) Bar chart showing the number of DEGs in each pairwise comparison. (F) Venn diagrams illustrating the overlap of DEGs.

To investigate global transcriptional profiles, heatmaps of differentially expressed genes (DEGs) were generated for four comparisons: DMD versus WT, BMD versus WT, BMD versus DMD (Figure S1A–C) and the integrated comparison of BMD versus DMD versus WT (Figure 1A). Hierarchical clustering showed that DMD samples consistently formed a distinct cluster, reflecting their pronounced molecular disruption, whereas BMD samples frequently grouped with WT, consistent with their milder phenotype and partially preserved transcriptomic profile. Notably, one BMD sample (BMD_3) clustered particularly close to WT samples, consistent with the intermediate nature and heterogeneity of the BMD phenotype. However, PCA (Figure S2) of the global mRNA and miRNA expression profiles demonstrates a clear segregation among the three groups. Within this context, BMD_3 remains closer to the WT cluster but does not constitute a distinct outlier within the BMD cohort. This minor variation is consistent with expected biological heterogeneity in porcine models and did not alter the overall transcriptomic and miRNA signatures distinguishing the three groups. The heatmaps further revealed condition‐specific clusters of upregulated and downregulated genes, underscoring the molecular heterogeneity underlying the progression of DMD and BMD. To further characterize transcriptional alterations at the individual gene level, volcano plots were generated for pairwise comparisons: DMD versus WT, BMD versus WT and BMD versus DMD (Figure 1B–D). A summary of DEG counts across comparisons is provided in Figure 1E. Relative to WT, 1440 genes were upregulated, and 487 genes were downregulated in DMD muscle, whereas 1804 genes were upregulated and 155 genes were downregulated in BMD muscle. In the direct comparison of BMD and DMD, 1475 genes were upregulated, and 1486 genes were downregulated in BMD muscle (Table S1).

To assess the overlap and divergence of transcriptional changes between groups, Venn diagram analyses were performed (Table S1). A total of 1282 genes were upregulated only in DMD, and 1646 were exclusively upregulated in BMD, whereas 158 genes were commonly upregulated in both conditions (Figure 1F). Similarly, 27 transcripts were commonly suppressed in both DMD and BMD, whereas 460 genes were suppressed only in DMD and 128 only in BMD relative to WT (Figure 1F). Cross‐comparisons further revealed opposite patterns of regulation between the two dystrophic models. Specifically, 17 genes were upregulated in DMD but downregulated in BMD, whereas 13 genes were downregulated in DMD and upregulated in BMD (Figure S1D). This analysis reveals distinct transcriptional signatures in DMD and BMD muscle compared to WT controls, highlighting the distinct molecular pathologies of these dystrophinopathies despite their genetic relationship. To validate the RNA‐seq findings, quantitative reverse transcription PCR (qRT‐PCR) was performed on triceps brachii samples from WT, *DMD*Δ52 (DMD) and *DMD*Δ51–52 (BMD) pigs following the protocol described by Jaudas et al. [21]. Expression of four inflammatory genes (*IL8*, *IL10*, *TGFB* and *TNFA*) was normalized to *GAPDH*. The qRT‐PCR results were consistent with the RNA‐seq data, confirming upregulation of *IL10* and *TGFB* in DMD, but not in BMD (Figure S3 and Table S1).

### 3.2 Enrichment Analysis of Genes Uniquely Downregulated in DMD Muscle

**FIGURE 2:**
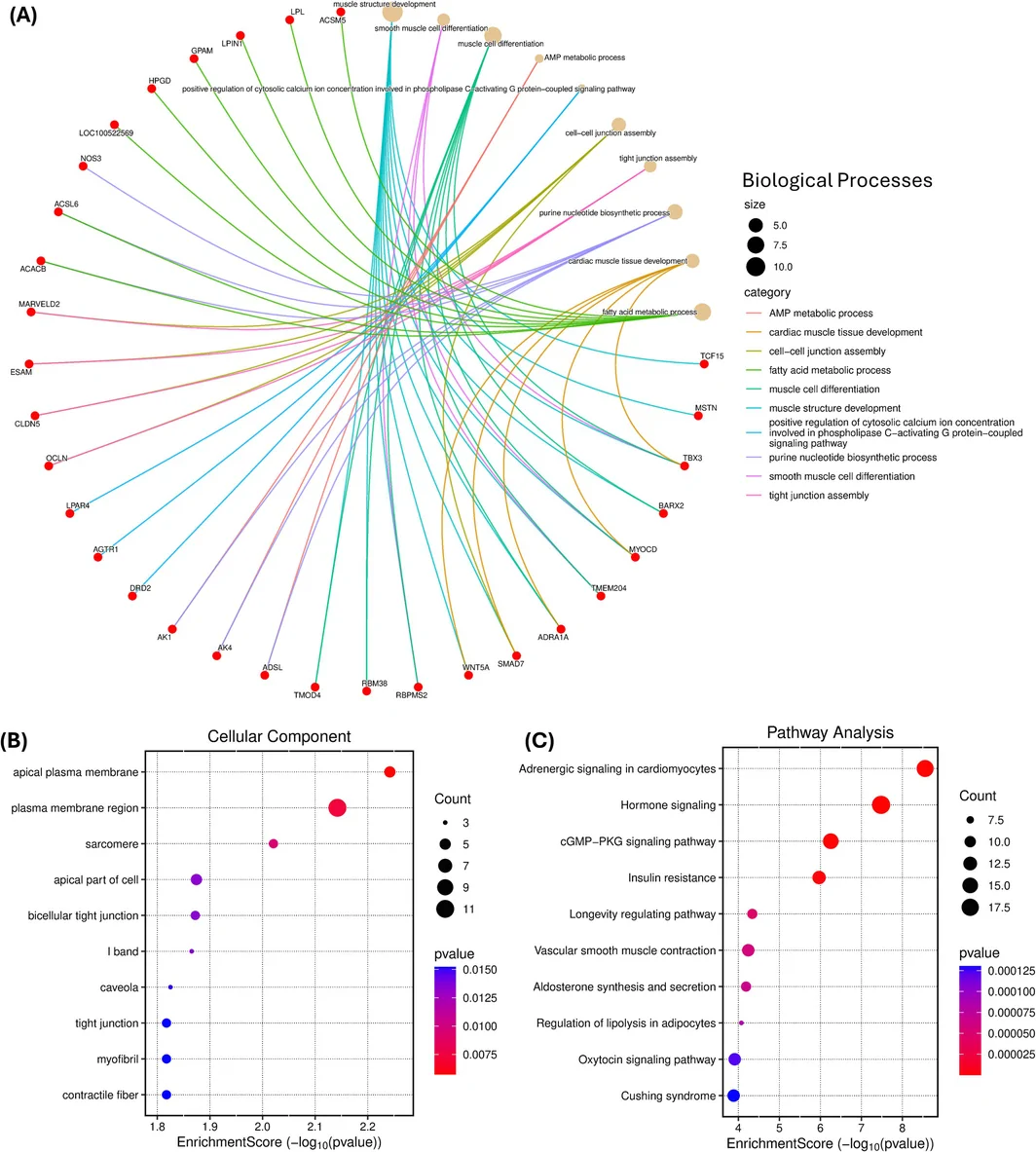
Enrichment analysis of genes uniquely downregulated in DMD muscle. (A) CNET plot showing the interconnected network of enriched BPs and their associated genes. Pathway nodes indicate functional categories, whereas gene nodes highlight shared contributions across multiple pathways. (B) Dot plot displaying the top 10 enriched CCs and (C) KEGG pathways. Dot size reflects the number of genes per pathway, and colour indicates statistical significance.

In the first step, GO and KEGG enrichment analyses were performed on 460 genes uniquely downregulated in DMD muscle, defined as genes downregulated only in DMD compared to WT, but unchanged in BMD compared to WT (Figure 1F). GO analysis categorized these genes into the three functional domains: biological processes (BP), cellular components (CC) and molecular functions (MF). In the BP category, the downregulated genes were enriched for muscle cell differentiation, muscle structure development, cardiac muscle tissue development and smooth muscle cell differentiation (Figure 2A). CC terms indicated enrichment in core structural components of muscle fibres, such as the contractile fibre, myofibril, sarcomere and I band, as well as junctional and membrane‐associated components, such as the tight junction and apical plasma membrane (Figure 2B). MF enrichment highlighted activities relevant to muscle homeostasis and signalling, including ligase activity, frizzled receptor binding, exopeptidase activity, tropomyosin binding and NADP binding (Figure S4A). KEGG pathway analysis further emphasized the functional relevance of these DMD‐specific downregulated genes, revealing significant enrichment in pathways central to muscle and metabolic regulation, including cytoskeletal organization in muscle cells, hormone signalling pathways, Cushing syndrome, insulin resistance and regulation of lipolysis in adipocytes (Figure 2C). All significantly enriched terms and pathways are listed in Table S2. Together, these enrichment analyses indicate that DMD‐specific transcriptional deficits predominantly affect pathways governing muscle differentiation, contractile function and metabolic regulation, likely contributing to impaired muscle development and disease progression in dystrophinopathy.

### 3.3 Enrichment Analysis of Genes Uniquely Upregulated in DMD Muscle

**FIGURE 3:**
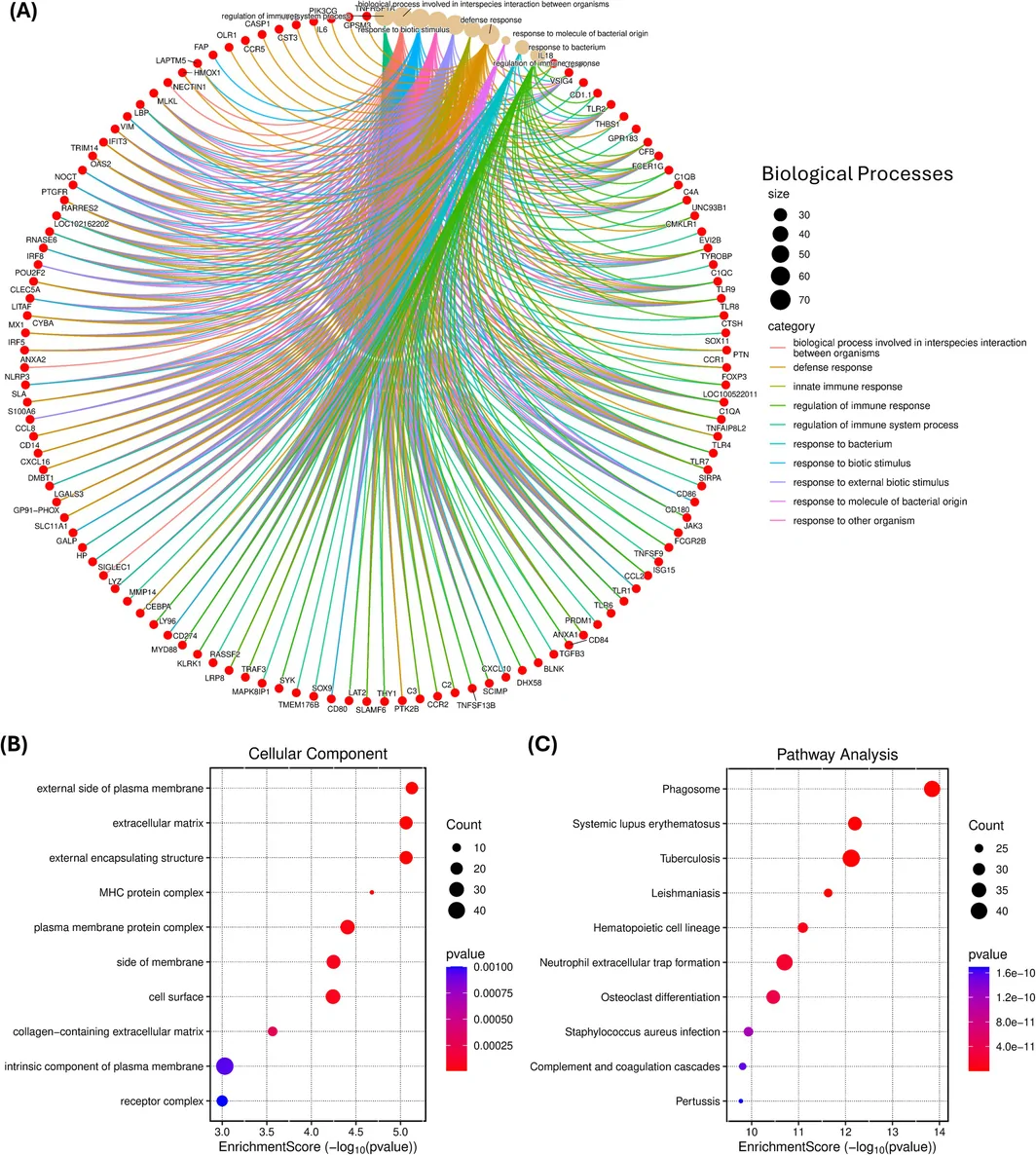
Enrichment analysis of genes uniquely upregulated in DMD muscle. (A) CNET plot showing the interconnected network of enriched BPs and their associated genes. (B) Dot plot displaying the top 10 enriched CCs and (C) KEGG pathways.

In addition to downregulated transcripts, we analysed 1282 genes uniquely upregulated in DMD muscle, defined as genes elevated in DMD compared with WT but unchanged in BMD (Figure 1F). GO enrichment analysis revealed significant overrepresentation of important BPs, including activation of the immune response, positive regulation of phagocytosis, cytokine production, actin cytoskeleton reorganization and complement activation (Figure 3A). CC enrichment analysis highlighted associations with the MHC protein complex, extracellular matrix, microtubule cytoskeleton and intrinsic component of plasma membrane (Figure 3B). MF enrichment highlighted cytoskeletal protein binding, immune receptor activity, actin filament binding, cytokine receptor activity and chemokine binding (Figure S4B). KEGG pathway analysis further supported the involvement of these upregulated genes in inflammation and signalling with significant enrichment in complement and coagulation cascades, osteoclast differentiation, cytoskeleton in muscle cells, cytokine–cytokine receptor interaction and chemokine signalling pathway (Figure 3C). All significantly enriched terms and pathways are listed in Table S3. Together, these findings suggest that transcriptional upregulation in DMD muscle extends beyond structural remodelling to encompass the broad activation of immune and signalling pathways, reflecting key molecular drivers of dystrophic pathology. Equivalent enrichment analyses for the 1646 uniquely upregulated and 128 uniquely downregulated genes in BMD muscle are summarized in Tables S4 and S5 (Figures S5 and S6).

### 3.4 Altered miRNA Profiles in DMD and BMD Muscle

**FIGURE 4:**
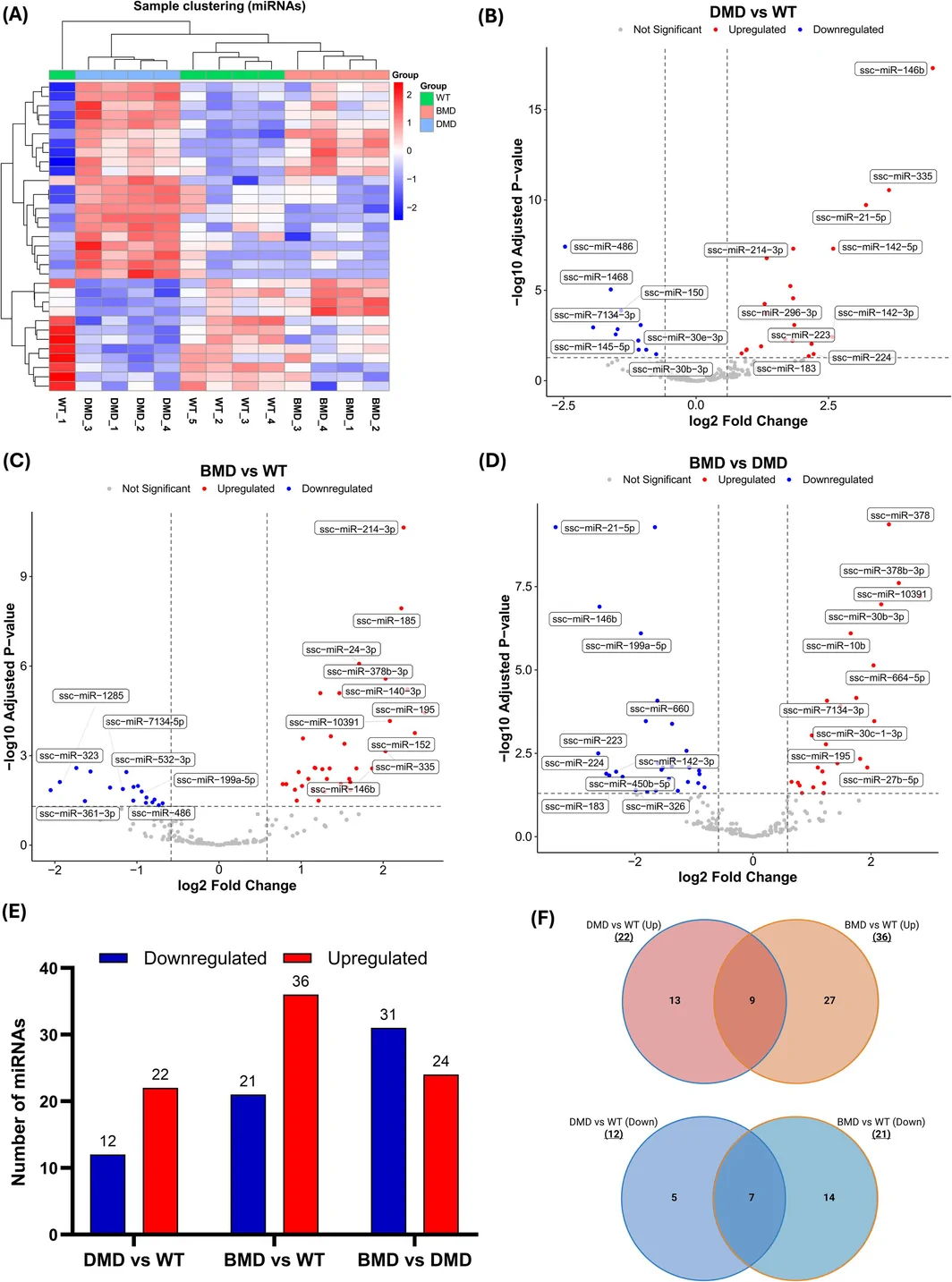
Differential expression analysis of miRNA. (A) Heatmaps of differentially expressed miRNAs across all groups. (B–D) Volcano plots of differentially expressed miRNAs in pairwise comparisons. (E) Bar chart showing the number of differentially expressed miRNAs in each pairwise comparison. (F) Venn diagrams illustrating the overlap of differentially expressed miRNAs.

A total of 457 miRNAs were detected, of which 34 (7.4%) were significantly dysregulated in DMD versus WT, 57 (12.5%) in BMD versus WT and 55 (12.0%) in BMD versus DMD (adjusted *p* ≤ 0.05). To assess global alterations in miRNA expression, heatmaps were generated for DMD versus WT, BMD versus WT and BMD versus DMD (Figure S7A–C), as well as for all three groups combined (Figure 4A). As observed for mRNA expression, DMD samples formed a distinct cluster, indicating widespread miRNA dysregulation, whereas BMD samples frequently grouped with WT, consistent with their intermediate phenotype. Clear clusters of up‐ and downregulated miRNAs were identified across conditions, indicating condition‐specific regulatory programmes that contribute to disease severity. To identify individual miRNAs underlying these patterns, volcano plots were generated (Figure 4B–D), and summary counts of differentially expressed miRNAs are shown in Figure 4E. Relative to WT, 22 miRNAs were upregulated and 12 were downregulated in DMD muscle, whereas BMD muscle exhibited 36 upregulated and 21 downregulated miRNAs. Direct comparison of BMD versus DMD revealed 24 upregulated and 31 downregulated miRNAs.

Overlap analysis defined shared and model‐specific miRNA signatures (Table S1). Among upregulated miRNAs, 13 were unique to DMD, 27 were unique to BMD, and nine were shared between both groups (Figure 4F). For downregulated miRNAs, five were specific to DMD, 14 to BMD, and seven were common to both models (Figure 4F). Cross‐comparisons identified a small set of inversely regulated miRNAs: one upregulated in DMD but downregulated in BMD and two downregulated in DMD but upregulated in BMD (Figure S7D).

Additionally, we performed a cross‐species and cross‐study replication analysis of the differentially expressed miRNAs identified in our dataset by comparison with two published human neuromuscular disease studies: (i) the circulating small RNA‐seq dataset from Kakouri et al. [16], which reports the top 20 differentially expressed miRNAs per disease across five neuromuscular disorders in human serum, and (ii) the miRNA microarray dataset from Eisenberg et al. [17], providing signed linear fold changes for 185 miRNAs in human muscle biopsies across 10 muscular disorders. This integrative analysis identified 35 miRNAs that were dysregulated in both the current porcine data and the previously published human data (Figure S8), with dysregulation of miR‐296‐3p and miR‐423‐5p emerging as novel candidate findings. Collectively, these results reveal both shared and distinct miRNA expression patterns in porcine models of dystrophinopathies, underscoring model‐specific post‐transcriptional regulatory mechanisms. Moreover, they highlight a subset of inversely regulated miRNAs that may delineate key molecular differences between DMD and BMD.

### 3.5 miRNAs and Their Target Genes

**FIGURE 5:**
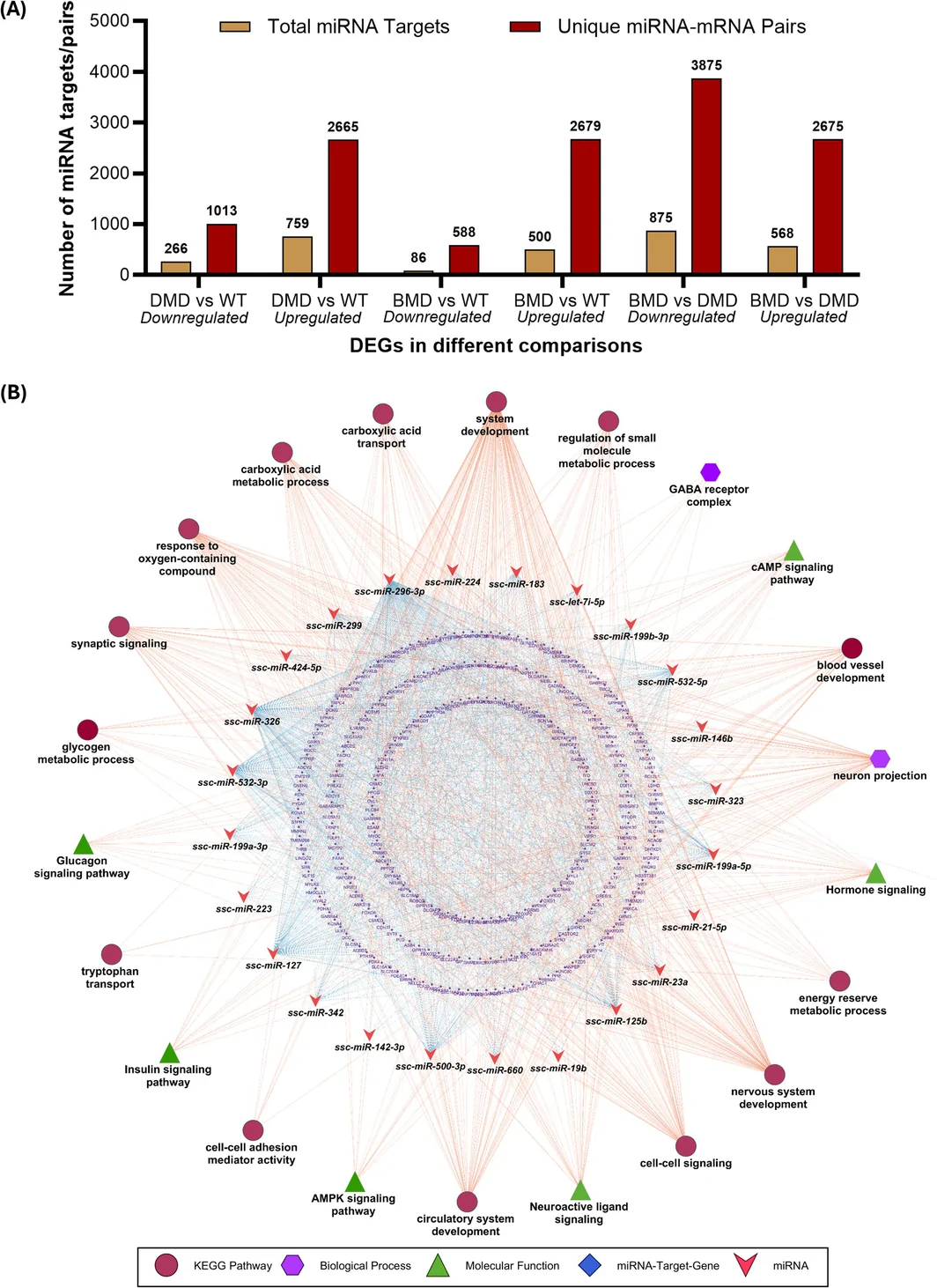
miRNA–mRNA networks. (A) Total number of genes negatively correlated with, and potentially targeted by, differentially expressed miRNAs, along with the corresponding number of unique miRNA–mRNA pairs for each group comparison. (B) Representative enriched biological processes and KEGG pathways for genes upregulated in BMD versus DMD, together with their associated downregulated miRNAs.

To identify pathways regulated by dysregulated miRNAs, we first identified all negatively correlated target DEGs of differentially expressed miRNAs across the different group comparisons. Because individual miRNAs can regulate multiple transcripts, and single genes can be targeted by several miRNAs, this analysis yielded extensive miRNA–mRNA interaction networks (Table S1). In the DMD versus WT comparison, 22 upregulated miRNAs were predicted to target 266 downregulated transcripts, resulting in 1013 unique miRNA–mRNA interactions. Conversely, 12 downregulated miRNAs were associated with 759 upregulated genes, forming 2665 unique pairs. In BMD versus WT, 36 upregulated miRNAs were predicted to target 86 downregulated genes, yielding 588 unique pairs, whereas 21 downregulated miRNAs were linked to 500 upregulated genes, yielding 2679 unique pairs. In the BMD versus DMD comparison, 24 upregulated miRNAs were associated with 875 downregulated target genes through 3875 unique interactions, whereas 31 downregulated miRNAs targeted 568 upregulated genes, yielding 2675 unique miRNA–mRNA pairs (Figure 5A).

To investigate the biological pathways regulated by differentially expressed miRNAs in the BMD versus DMD comparison and thereby assess molecular pathways influenced by exon skipping, we conducted GO and KEGG enrichment analyses. First, we analysed miRNAs upregulated in BMD relative to DMD and their negatively correlated target genes. This analysis identified 86 downregulated DEGs predicted to be targets of 30 upregulated miRNAs (Table S1). Enrichment analyses revealed significant associations with pathways relevant to dystrophic pathology, including chemokine signalling pathway, phagosome, neurogenesis, cellular component organization, fat cell differentiation, osteoclast differentiation, apoptotic process and chemotaxis (Figure S9). All significantly enriched terms and pathways are listed in Table S6.

Complementing this analysis, we examined miRNAs downregulated in BMD relative to DMD and their negatively correlated targets. This analysis identified 500 upregulated genes as predicted targets of 21 downregulated miRNAs. Enrichment analysis of these genes revealed significant involvement in biological processes and pathways associated with dystrophinopathy, including system and vascular development, insulin and glucagon signalling, synaptic and neuronal signalling, neuron projection organization, tryptophan transport and multiple metabolic pathways (Figure 5B). All significantly enriched terms and pathways are listed in Table S7. These results suggest that changes in miRNA–mRNA regulation following exon skipping affect multiple developmental, metabolic and signalling pathways, potentially contributing to the partial phenotypic rescue observed in the BMD‐like model.

Building on these findings, we performed an integrative analysis of significantly upregulated miRNAs to evaluate their potential as biomarkers or therapeutic targets in DMD and BMD. Sixteen miRNAs were specifically upregulated in DMD relative to both BMD and WT, whereas 18 miRNAs were upregulated in BMD relative to both WT and DMD, highlighting disease‐specific miRNA signatures. The 16 DMD‐associated miRNAs were predicted to target 246 negatively correlated DEGs, whereas the 18 BMD‐associated miRNAs targeted 80 negatively correlated DEGs (Table S1). These disease‐specific miRNAs represent promising non‐invasive diagnostic biomarkers and potential therapeutic targets, with the full list of miRNAs and their respective target gene counts provided in Table 1.

**TABLE 1:** Potential biomarker miRNAs and their negatively correlated target genes.

DMD‐specific miRNAs (upregulated in DMD vs. both BMD and WT)		BMD‐specific miRNAs (upregulated in BMD vs. both DMD and WT)	
miRNAs (16)	Number of downregulated target genes	miRNAs (18)	Number of downregulated target genes
ssc‐miR‐296‐3p	228	ssc‐miR‐423‐5p	67
ssc‐miR‐199a‐5p	77	ssc‐miR‐30c‐1‐3p	48
ssc‐miR‐183	47	ssc‐miR‐30b‐3p	39
ssc‐miR‐199a‐3p	38	ssc‐miR‐664–5p	34
ssc‐miR‐660	30	ssc‐miR‐378	27
ssc‐let‐7i‐5p	26	ssc‐let‐7d‐5p	21
ssc‐miR‐23a	25	ssc‐miR‐10 391	20
ssc‐miR‐199b‐3p	22	ssc‐miR‐140‐3p	15
ssc‐miR‐224	22	ssc‐let‐7a	12
ssc‐miR‐223	19	ssc‐let‐7 g	12
ssc‐miR‐424–5p	15	ssc‐miR‐378b‐3p	11
ssc‐miR‐142‐3p	12	ssc‐miR‐143‐3p	10
ssc‐miR‐146b	9	ssc‐let‐7f‐5p	6
ssc‐miR‐21‐5p	8	ssc‐miR‐10b	6
ssc‐miR‐542‐3p	2	ssc‐miR‐26a	6
ssc‐miR‐142‐5p	0	ssc‐miR‐195	5
		ssc‐miR‐133a‐5p	1
		ssc‐miR‐1	0

**FIGURE 6:**
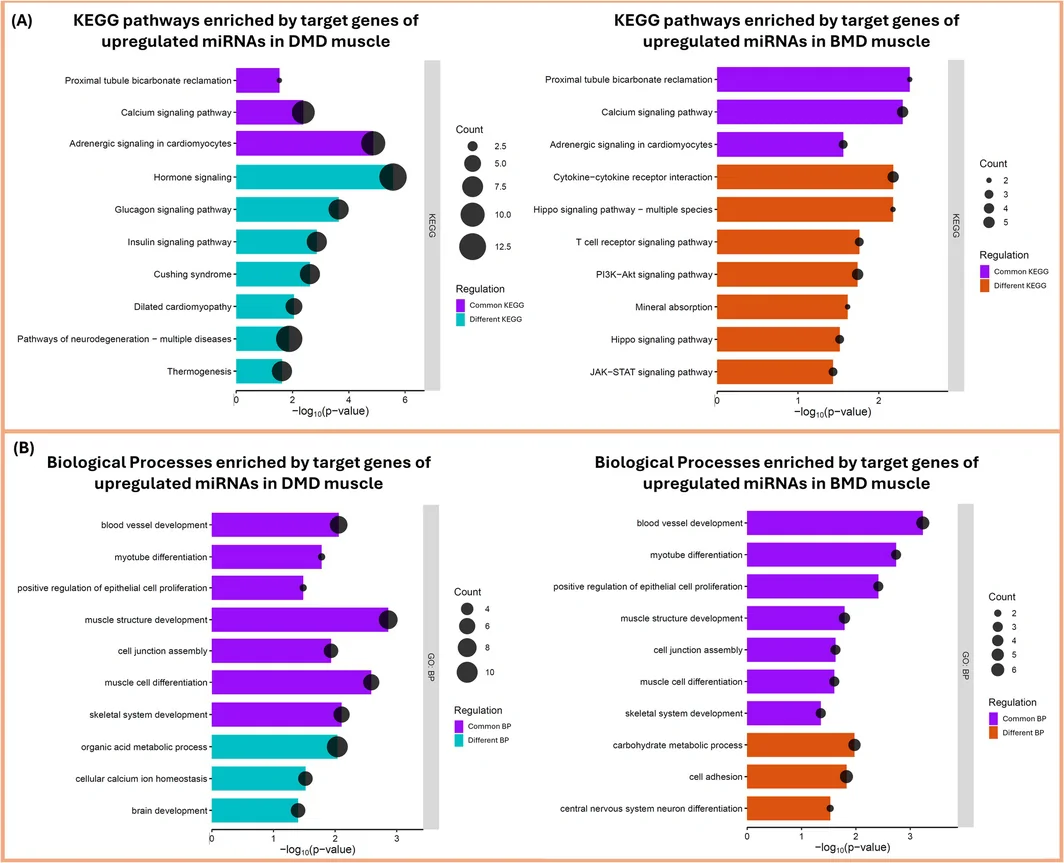
Enrichment analyses of downregulated target genes of upregulated miRNAs in DMD and BMD skeletal muscles. Dot size represents the number of genes contributing to each pathway, and dot colour indicates whether the pathway is enriched in both BMD and DMD or uniquely in one condition. (A) KEGG pathway enrichment showing three shared and seven condition‐specific pathways. (B) Biological process enrichment showing seven shared and three condition‐specific terms.

Enrichment analyses of downregulated target genes of upregulated miRNAs specific to DMD and BMD muscles revealed distinct molecular signatures between the two conditions. KEGG pathway analysis revealed that pathways implicated in muscular dystrophy pathogenesis, including Cushing syndrome, insulin signalling pathway, WNT signalling pathway and thyroid hormone synthesis, were enriched exclusively in DMD muscles. In contrast, pathways such as calcium signalling and adrenergic signalling in cardiomyocytes were enriched in both DMD and BMD, with a greater number of associated genes in DMD (Figure 6A), supporting the notion of a more pronounced dysregulation in the more severe phenotype. BP enrichment displayed a similar pattern (Figure 6B). All significantly enriched terms and pathways are listed in Table S8.

**FIGURE 7:**
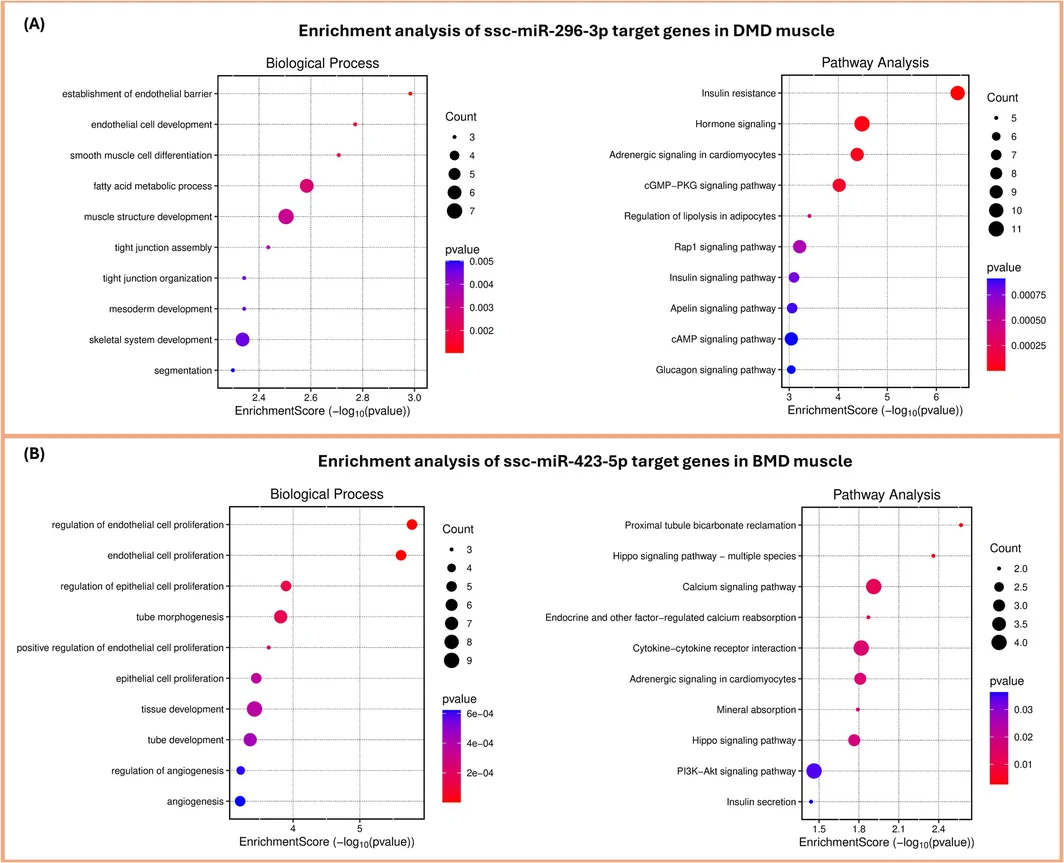
Enrichment analyses of downregulated target genes of (A) ssc‐miR‐296‐3p in DMD muscle and (B) ssc‐miR‐423‐5p in BMD muscle.

Among the upregulated miRNAs with potential biomarker value, two emerged as particularly significant: ssc‐miR‐296‐3p and ssc‐miR‐423‐5p. ssc‐miR‐296‐3p was upregulated exclusively in DMD muscle and predicted to target 228 downregulated genes, whereas ssc‐miR‐423‐5p was upregulated only in BMD muscle and targeted 67 downregulated genes. Enrichment analysis revealed that ssc‐miR‐296‐3p targets were involved in muscle structure development, skeletal system development, fatty acid metabolism and hormone signalling (Figure 7A), suggesting a direct contribution to DMD pathogenesis. In contrast, ssc‐miR‐423‐5p targets were enriched in endothelial cell proliferation, tissue development, calcium signalling and cytokine–cytokine receptor interaction (Figure 7B), reflecting a broader role in tissue and vascular homeostasis rather than direct regulation of skeletal muscle. All significantly enriched terms and pathways are listed in Table S9. These results indicate a direct link between miRNA dysregulation and dystrophic progression, with ssc‐miR‐296‐3p potentially driving DMD pathology and ssc‐miR‐423‐5p contributing to the milder phenotype observed in BMD.

## 4 Discussion

This study utilized porcine models to investigate transcriptomic alterations in dystrophinopathies. The *DMD*Δ52 model recapitulates human DMD, whereas the *DMD*Δ51–52 model mimics exon‐skipping–mediated therapeutic correction and resembles a BMD‐like phenotype. Exon 51 skipping can restore the reading frame in approximately 14% of DMD patients, particularly those with deletions involving exon 50 or 52 [22]. In the porcine model, the constitutive genomic deletion provides uniform reframing across all muscle fibres, representing an upper bound of molecular rescue [23]. In contrast, FDA‐approved exon‐skipping therapies typically achieve only low and highly variable levels of dystrophin restoration in patients [22]. Nevertheless, detailed molecular profiling of the DMD and BMD‐like porcine models provides valuable insights into the potential of exon‐skipping strategies to reshape the skeletal muscle transcriptome. The triceps brachii muscle was chosen for transcriptomic and miRNA analysis because it exhibits consistent and well‐characterized dystrophic pathology in the *DMD*Δ52 model at 3.5 months of age, whereas *DMD*Δ51–52 muscle shows only minor changes. As a readily accessible weight‐bearing muscle, it enables standardized biopsy collection under identical conditions, thereby facilitating reliable inter‐genotype comparisons and direct correlation with previously published histopathological and functional data from the same muscle.

Key genes involved in calcium homeostasis (*CACNA2D3*, *CACNA2D4*), skeletal muscle development and function (*MYBPC2*, *MYL3*, *MYLK2*), cardiac/smooth muscle regulation (*MYH11*, *MYLK3*, *MYOCD*) and cytoskeletal organization (*ATP1B2*, *MYH11*, *FHL3*, *MYL3*, *TLN2*, *DMD*, *COL6A5*, *MYBPC2*, *TMOD4*) were downregulated exclusively in DMD muscle. Conversely, pro‐inflammatory mediators (*IL6*, *IL18*, *IL1R1*), chemokine receptors (*CCR1/2/5/6*), Toll‐like receptors (*TLR1/2/4/6/7/8/9*) and several other inflammatory regulators (*NLRP1*, *NLRP3*, *TNFSF9/15/18*) were upregulated only in DMD muscle. These findings illustrate the convergence of cytoskeletal dysfunction, immune activation and systemic inflammation in DMD pathophysiology. In the BMD model, muscle dysfunction–related transcriptomic changes were less severe than in DMD, showing partial restoration of structural, contractile, metabolic and inflammatory pathways. Overall, fewer genes were enriched for terms associated with muscle dysfunction in the BMD group than in the DMD group. This molecular rescue aligned well with our recently published functional data showing severely reduced muscle force in DMD pigs and partial, yet incomplete, recovery in BMD animals [13]. Enrichment of terms related to cardiac muscle tissue development among downregulated genes in both BMD and DMD skeletal muscle largely reflects the repression of genes encoding shared contractile and cytoskeletal proteins expressed in both striated muscle types. These alterations are consistent with the well‐documented cardiomyopathy in dystrophinopathies [24].

Beyond these transcriptomic alterations, miRNA‐mediated post‐transcriptional regulation represents an additional layer of control in the pathogenesis of dystrophinopathy [25, 26]. We identified both shared and condition‐specific miRNA signatures that targeted numerous inversely correlated DEGs. Among the DMD‐specific miRNAs, miR‐296‐3p emerged as a novel candidate predicted to target 228 downregulated genes enriched in muscle and skeletal system development, tight‐junction assembly, and hormone signalling. As miR‐296‐3p has not been directly linked to human muscular dystrophies, it can serve as a pig‐specific biomarker for DMD. Two miRNAs of particular interest, miR‐199a‐5p and miR‐199b, were strongly upregulated in DMD pig muscle. miR‐199a‐5p was significantly elevated in DMD compared with both WT and BMD. Previous studies have linked miR‐199a‐5p dysregulation to dystrophic muscle pathogenesis and demonstrated its upregulation in exosomes derived from DMD muscle fibroblasts [27, 28]. In the present dataset, miR‐199a‐5p was predicted to target 77 downregulated genes in DMD muscle. Similarly, miR‐199b, which is known to repress porcine muscle satellite cell proliferation via a JAG1‐NOTCH1 feedback loop [29], was predicted to target 22 downregulated genes in this study. Cross‐comparison with published human data revealed that both miR‐199a‐5p and miR‐199b were upregulated in skeletal muscle from patients with various forms of muscular dystrophy (Figure S8 [16, 17]). Given their established roles in muscle development and dystrophy, together with the substantial number of predicted targets in our dataset, we speculate that miR‐199a‐5p and miR‐199b represent promising biomarkers for monitoring disease progression and therapeutic efficacy, including exon‐skipping interventions, in both porcine models and human patients. Other DMD‐specific miRNAs, including miR‐660, let‐7i‐5p, miR‐424‐5p and miR‐142‐3p, have known associations with muscle dysfunction and signalling dysregulation [30, 31, 32, 33, 34]. Interestingly, miR‐199a‐3p, miR‐23a, miR‐223, miR‐146b and miR‐224, which are known for their protective or anti‐atrophic effects [35, 36, 37, 38, 39], were also upregulated, possibly reflecting compensatory feedback mechanisms against muscle wasting and fibrosis in DMD.

Interestingly, a greater number of miRNAs were dysregulated in BMD versus WT than in DMD versus WT. This may reflect ongoing regeneration and remodelling in BMD muscle, whereas advanced degeneration and fibro‐adipose replacement in DMD could reduce the complexity of active miRNA regulation. miR‐423‐5p was specifically upregulated in the BMD muscle, a novel finding that could serve as a biomarker of BMD in the pig model. Its role in muscle growth and development [40] supports its diagnostic and therapeutic potential; however, it has never been directly linked with BMD or DMD. It can potentially target 67 downregulated genes involved in angiogenesis, tissue development, calcium signalling and insulin secretion. miR‐664‐5p promotes early myoblast proliferation but inhibits later differentiation by targeting *SRF* and *WNT1* [41]. miR‐378 enhances myogenic regeneration by repressing MYOR, an antagonist of MYOD [42], although its sustained expression exacerbates dystrophic pathology, whereas inhibition improves muscle function [43]. Thus, the elevated levels of miR‐664‐5p and miR‐378 in BMD may sustain myoblast proliferation and partially preserve regenerative capacity, contributing to the milder clinical phenotype. In contrast, members of the miR‐30 family promote myoblast differentiation and muscle formation [44]. Their upregulation in BMD may represent a compensatory mechanism that counteracts pathways impairing muscle growth and function.

Cross‐species comparison revealed substantial overlap between the porcine DMD miRNA signature and that of human DMD muscle, as well as other forms of muscular dystrophy, supporting shared molecular mechanisms (Figure S8 [16, 17]). Although serum samples from the animals in this study were not profiled for miRNAs, the skeletal muscle miRNA signature in humans is also not reflected in the serum (Figure S8 [16, 17]). It can be speculated that not all miRNA dysregulation in skeletal muscle is reflected in circulating blood. Moreover, skeletal muscle biopsy analysis is a feasible and informative approach for transcriptomic profiling in dystrophic muscle.

Although porcine models involve higher costs and more limited accessibility compared to the widely used *mdx* mouse, they offer important complementary advantages for translational DMD research. The *mdx* mouse exhibits only a mild phenotype, with robust muscle regeneration and minimal cardiac involvement, limiting its predictive value for human disease progression [45, 46, 47]. The Golden Retriever muscular dystrophy (GRMD) dog model, in contrast, more closely recapitulates the severe, progressive skeletal muscle degeneration, fibrosis and cardiomyopathy seen in human DMD and has been instrumental for preclinical testing of gene therapies and for identifying potential immunological complications [48]. However, GRMD dogs show considerable phenotypic variability even within litters, are limited by long generation intervals, seasonal breeding and relatively small litter sizes, which constrain the generation of statistically robust cohort sizes [49].

The *DMD*Δ52 (DMD) and *DMD*Δ51–52 (BMD‐like) pig models closely recapitulate key pathological features of human dystrophinopathies, including progressive myofiber degeneration, fibrosis, inflammation and cardiac involvement, in an accelerated yet highly consistent manner that closely mirrors the severity and timeline of human DMD. Their larger body size enables realistic testing of dosing regimens, delivery routes and repeated longitudinal biopsies, whereas genetic tailoring to the common human exon 52 deletion hotspot and the production of larger, genetically homogeneous cohorts provide clear practical advantages for omics studies and therapeutic validation. The earlier proteome analysis of the *DMD*Δ52 pig muscle revealed progressive, age‐dependent downregulation of structural, contractile and metabolic proteins, accompanied by upregulation of inflammatory, extracellular matrix and stress‐response pathways [50]. The transcriptional repression of sarcomeric and contractile genes (e.g., *MYBPC2*, *MYL3*, *MYLK2*) and activation of immune pathways (e.g., *IL6*, *IL18*, *TLRs*, *CCRs*) observed here parallel those proteomic changes and are largely consistent with findings reported in other dystrophinopathy models [51, 52]. Moreover, previous studies using these porcine models have successfully validated non‐invasive imaging biomarkers (e.g., multispectral optoacoustic tomography for muscle fibrosis) and in vivo CRISPR/Cas9‐mediated genome‐editing approaches [4, 11, 12, 13, 19, 50], underscoring their value in bridging proof‐of‐concept studies in rodent models and human therapeutic development.

Limitations of this study include the use of whole‐muscle biopsies, which may partly reflect shifts in cellular composition, such as increased inflammatory infiltration in DMD. This is supported by the strong upregulation of immune‐related genes and pathways. Consequently, attribution of specific changes exclusively to myofibers is limited, and future single‐nucleus RNA‐seq studies will help deconvolute cell type–specific contributions. In addition, miRNA–mRNA interactions were inferred from computational predictions and inverse correlations; functional validation will be required to establish causality. Moreover, it remains unclear whether the observed alterations in miRNA expression represent causal drivers of disease progression or are secondary consequences of ongoing muscle degeneration, which can be dissected through functional studies. Nevertheless, the present dataset offers valuable hypotheses and candidate miRNAs for further investigation in dystrophic muscle.

In conclusion, restoration of the dystrophin reading frame in the *DMD*Δ51–52 model partially reinstates structural, metabolic and signalling pathways disrupted in DMD, although not to WT levels. These findings validate the molecular and translational value of the porcine dystrophinopathy models for evaluating exon‐skipping therapies. The identification of condition‐specific miRNAs further provides insights into post‐transcriptional regulatory mechanisms that distinguish DMD from BMD and highlights promising biomarkers for disease stratification and therapeutic monitoring.

## Funding

This study was funded by the Bayerische Forschungsstiftung (AZ 802‐08; PDOC‐90‐15), the Else Kröner‐Fresenius‐Stiftung (EKFS; 2015_180) and the ForTra gGmbH für Forschungstransfer der EKFS (2018_T20; 2022_EKSE.41) and—in part—by the European Research Council (ERC, 101021043) and by the Leducq Foundation (Network 23CVD01). S.R. was supported by a doctoral scholarship (Promotionsstipendium) from the Friedrich‐Ebert‐Stiftung (FES).

## Conflicts of Interest

The authors declare no conflicts of interest.

## Supporting information


**Figure S1:** Differential gene expression analysis in skeletal muscle from DMD, BMD, and WT pigs. Heatmaps of differentially expressed genes (DEGs) in pairwise comparisons: (A) DMD versus WT, (B) BMD versus WT and (C) BMD versus DMD. Each column represents a biological replicate (*n* = 4–5 per group), and each row corresponds to a DEG (|log2FC| ≥ 1, adjusted *p* ≤ 0.05). Expression values were z‐transformed, with blue indicating lower expression and red indicating higher expression. The colour bar at the top denotes the experimental groups: WT (green), BMD (red) and DMD (blue). Hierarchical clustering highlights distinct expression patterns across genotypes. (D) Comparison of upregulated genes in DMD versus WT, with downregulated genes in BMD versus WT, and the comparison of downregulated genes in DMD versus WT, with upregulated genes in BMD versus WT.
**Figure S2:** Principal component analysis (PCA) of (A) mRNA and (B) miRNA expression profiles. The PCA plot of variance‐stabilized gene expression data, showing the first two principal components. Samples are coloured according to group: wild type (WT, green), Becker muscular dystrophy (BMD, red) and Duchenne muscular dystrophy (DMD, blue). Each point represents an individual sample.
**Figure S3:** Independent validation of RNA‐seq data by qRT‐PCR. Relative mRNA expression of selected inflammatory genes (*IL8*, *IL10*, *TGFB* and *TNFA*) was quantified by qRT‐PCR in an independent set of triceps brachii muscle samples from wild‐type (WT), *DMD*Δ52 (DMD) and *DMD*Δ51–52 (BMD) pigs (*n* = 4–5 per group). Expression levels were normalized to the housekeeping gene *GAPDH*. Statistical significance was assessed by a t‐test (*p < 0.05). The qRT‐PCR results closely corroborate the differential expression patterns observed in the RNA‐seq analysis.
**Figure S4:** (A) Dot plot displaying the top 10 enriched molecular functions by the genes uniquely downregulated in DMD skeletal muscle. (B) Dot plot displaying the top 10 enriched molecular functions by the genes uniquely upregulated in DMD skeletal muscle. Dot size reflects the number of genes per pathway, and colour indicates statistical significance.
**Figure S5:** Enrichment analysis of genes uniquely upregulated in BMD muscle. (A) CNET plot showing the interconnected network of enriched BPs and their associated genes. Pathway nodes indicate functional categories, while gene nodes highlight shared contributions across multiple pathways. (B) Dot plot displaying the top 10 enriched CCs and (C) KEGG pathways. Dot size reflects the number of genes per pathway, and colour indicates statistical significance.
**Figure S6:** Enrichment analysis of genes uniquely downregulated in BMD muscle. (A) CNET plot showing the interconnected network of enriched BPs and their associated genes. Pathway nodes indicate functional categories, while gene nodes highlight shared contributions across multiple pathways. (B) Dot plot displaying the top 10 enriched CCs and (C) KEGG pathways. Dot size reflects the number of genes per pathway, and colour indicates statistical significance.
**Figure S7:** Differential miRNA expression analysis in skeletal muscle from DMD, BMD and WT pigs. Heatmaps of differentially expressed miRNAs in pairwise comparisons: (A) DMD versus WT, (B) BMD versus WT and (C) BMD versus DMD. Each column represents a biological replicate (*n* = 4–5 per group), and each row corresponds to a differentially expressed miRNA (|log2FC| ≥ 1, adjusted *p* ≤ 0.05). Expression values were z‐transformed, with blue indicating lower expression and red indicating higher expression. The colour bar at the top denotes the experimental groups: WT (green), BMD (red) and DMD (blue). Hierarchical clustering highlights distinct expression patterns across genotypes. (D) Comparison of upregulated miRNAs in DMD versus WT, with downregulated miRNAs in BMD versus WT, and the comparison of downregulated miRNAs in DMD versus WT, with upregulated miRNAs in BMD versus WT.
**Figure S8:** Supplementary Figure S7: Cross‐species and cross‐studies comparison of differentially expressed miRNAs in the current study. Heatmap of log2 fold changes for 35 miRNAs (rows) detected as significantly differentially expressed (DE) in at least one of the samples analysed (columns) by Kakouri et al. (2022) or Eisenberg et al. (2007). The analysis spnas three independent studies: the current porcine skeletal muscle dataset (current study; two contexts: porcine DMD, porcine BMD), the human serum miRNA profile of Kakouri et al. (five neuromuscular diseases: DMD, DM1, DM2, FSHD1, LGMD‐R1; top‐20 DE miRNAs per disease) and the human muscle biopsy miRNA microarray dataset of Eisenberg et al. (ten neuromuscular disorders: DMD, BMD, LGMD2A, LGMD2B, FSHD, Miyoshi, NM, DM, PM, IBM). The upper annotation strip indicates the study origin (current study/Kakouri et al./Eisenberg et al.); the lower annotation strip distinguishes the analyte pool (M = muscle tissue, S = serum). Tile colour encodes log2FC clipped to [−5, +5] (blue = downregulated, white = 0, red = upregulated) to keep individual outliers from dominating the diverging scale; the number inside each tile shows the unclipped log2FC. Rows are ordered by number of contexts with a DE signal (most frequently replicated miRNAs at the top). Disease abbreviations are expanded beneath the heatmap. miRNA identifiers were harmonized to a species‐agnostic core name by removing the ssc−/has‐ prefix. For strand resolution, the 3p/5p suffix was retained where annotated; because the 2007 Eisenberg microarray platform did not distinguish strands, its entries were matched at the suffix‐stripped name level. Eisenbergs signed linear fold changes were converted to log2 fold changes according to log2FC = sign (fold) × log2(|fold|). No new statistical test was applied across studies. Every entry in the replication map represents a significant DE call by the threshold of the original study. This comparison identified 35 miRNAs that we reproducibly dysregulated across species and analyte pools.
**Figure S9:** Functional enrichment analysis of upregulated miRNAs in BMD relative to DMD and their downregulated target genes. A total of 86 downregulated DEGs, identified as targets of 30 upregulated miRNAs, were subjected to enrichment analysis. The figure illustrates representative enriched terms and pathways, along with the associated miRNAs. Terms and pathways shown passed the significance threshold of *p* ≤ 0.05. A complete list of significantly enriched terms and pathways associated with this gene set is provided in Table S6.

**Table S1:** Supporting Information.

**Table S2:** Supporting Information.

**Table S3:** Supporting Information.

**Table S4:** Supporting Information.

**Table S5:** Supporting Information.

**Table S6:** Supporting Information.

**Table S7:** Supporting Information.

**Table S8:** Supporting Information.

**Table S9:** Supporting Information.

## Data Availability

Data and materials are available upon request from the corresponding authors.
