# Supplementary material for: MicroRNA–mRNA Networks in Skeletal Muscle of Tailored Pig Models for Dystrophinopathies
Source: J Cachexia Sarcopenia Muscle. 2026 Sep 7;17(5):e70337. doi: 10.1002/jcsm.70337 (PMC13551010; doi:10.1002/jcsm.70337)
Supplement: Supplementary file 1 [file JCSM-17-e70337-s003.pdf]

Supplementary Figures for

MicroRNA-mRNA Networks in Skeletal Muscle of Tailored Pig Models for Dystrophinopathies

Sarah Reschke, Alexander Graf, Frieder Hadlich, Florian Jaudas, Thomas Fröhlich, Michael Stirm, Nikolai Klymiuk, Stefan Krebs, Eckhard Wolf, Asghar Ali

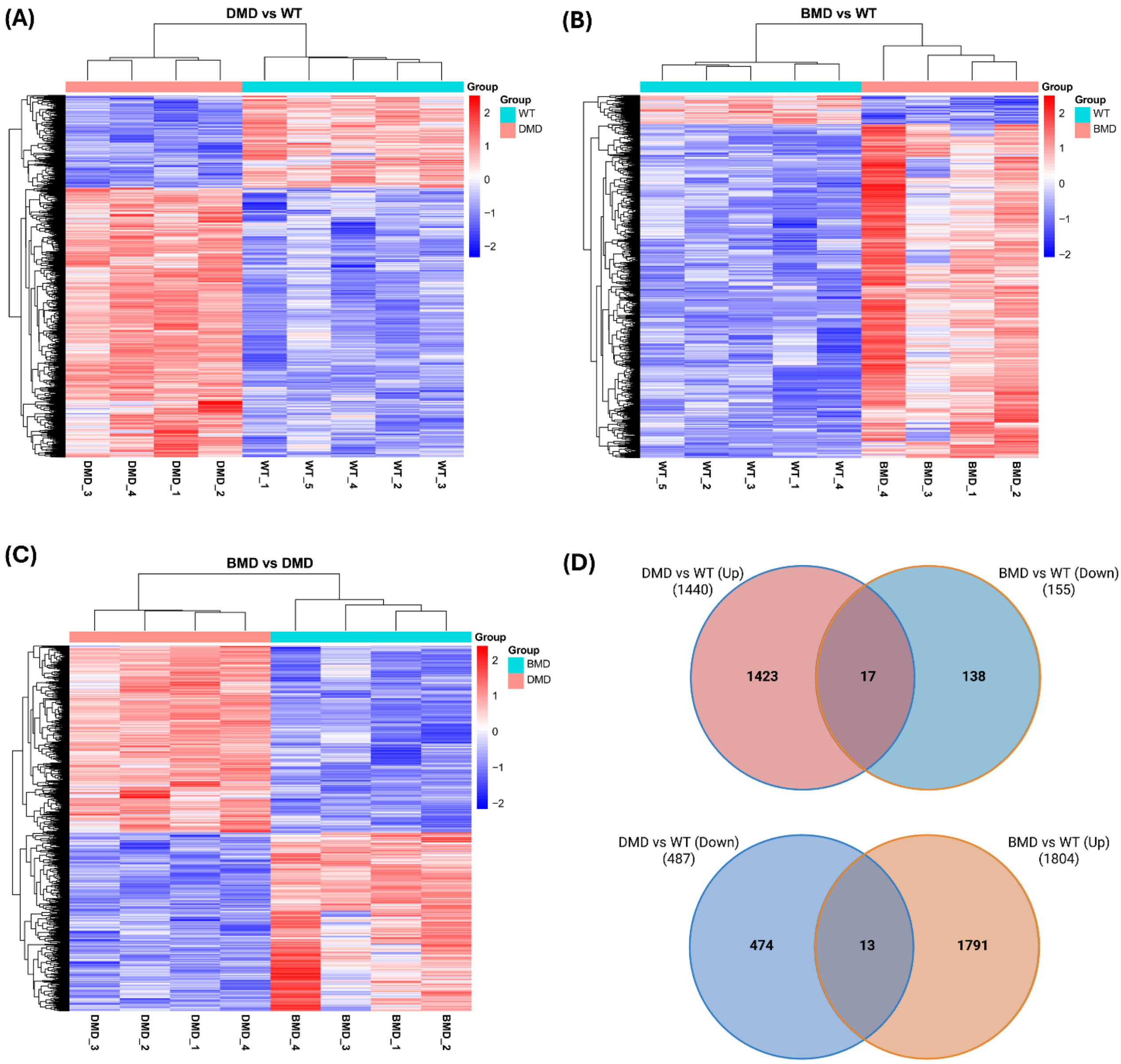

Fig. S1 Differential gene expression analysis in skeletal muscle from DMD, BMD, and WT pigs. Heatmaps of differentially expressed genes (DEGs) in pairwise comparisons: (A) DMD versus WT, (B) BMD versus WT, and (C) BMD versus DMD. Each column represents a biological replicate (n=4-5 per group), and each row corresponds to a DEG (|log2FC| ≥ 1, adjusted p-value ≤ 0.05). Expression values were z-transformed, with blue indicating lower expression and red indicating higher expression. The color bar at the top denotes the experimental groups: WT (green), BMD (red), and DMD (blue). Hierarchical clustering highlights distinct expression patterns across genotypes. (D) Comparison of upregulated genes in DMD versus WT, with downregulated genes in BMD versus WT, and the comparison of downregulated genes in DMD versus WT, with upregulated genes in BMD versus WT.

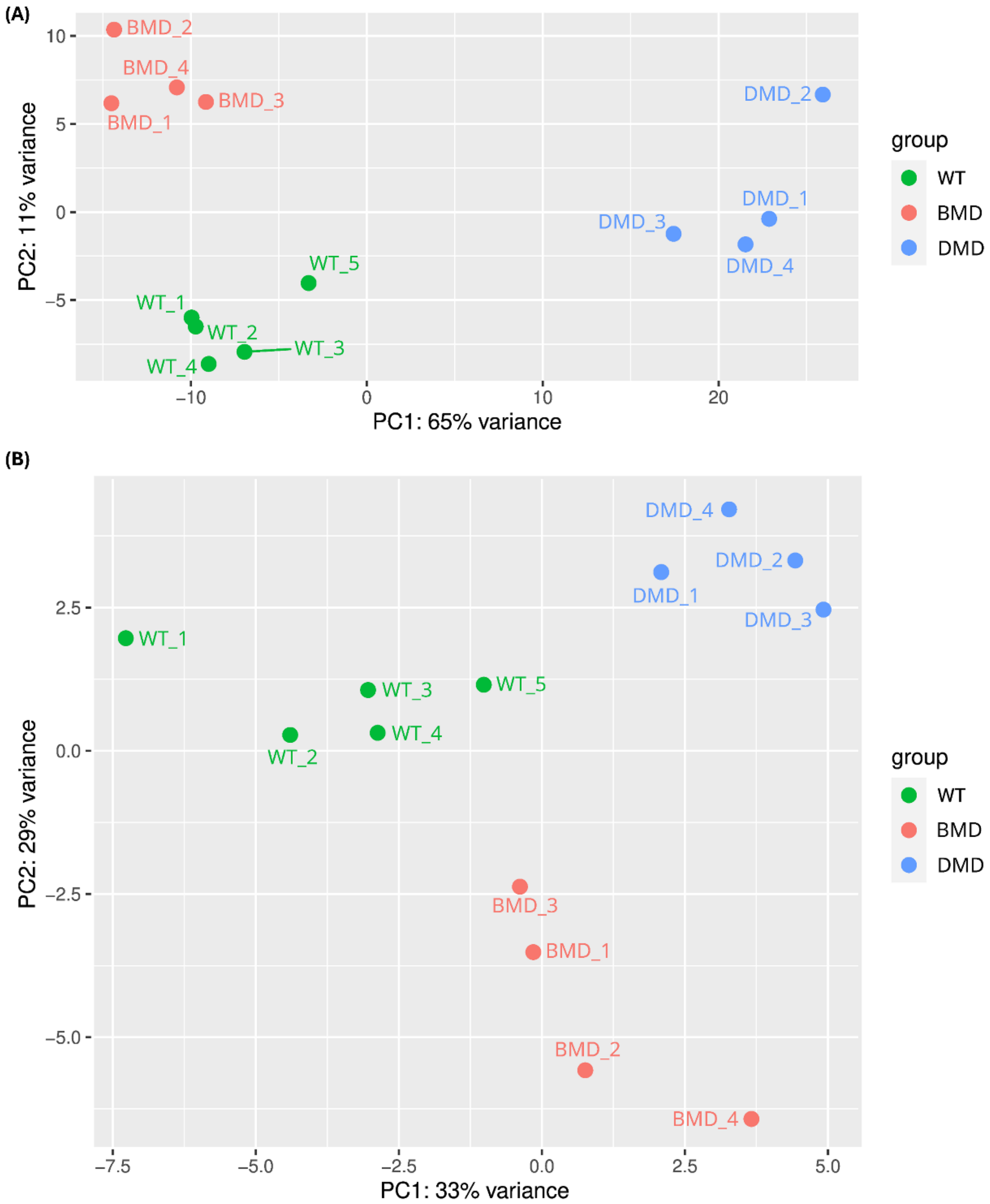

Figure S2: Principal component analysis (PCA) of (A) mRNA and (B) miRNA expression profiles. The PCA plot of variance-stabilized gene expression data, showing the first two principal components. Samples are colored according to group: wild type (WT, green), Becker muscular dystrophy (BMD, red), and Duchenne muscular dystrophy (DMD, blue). Each point represents an individual sample.

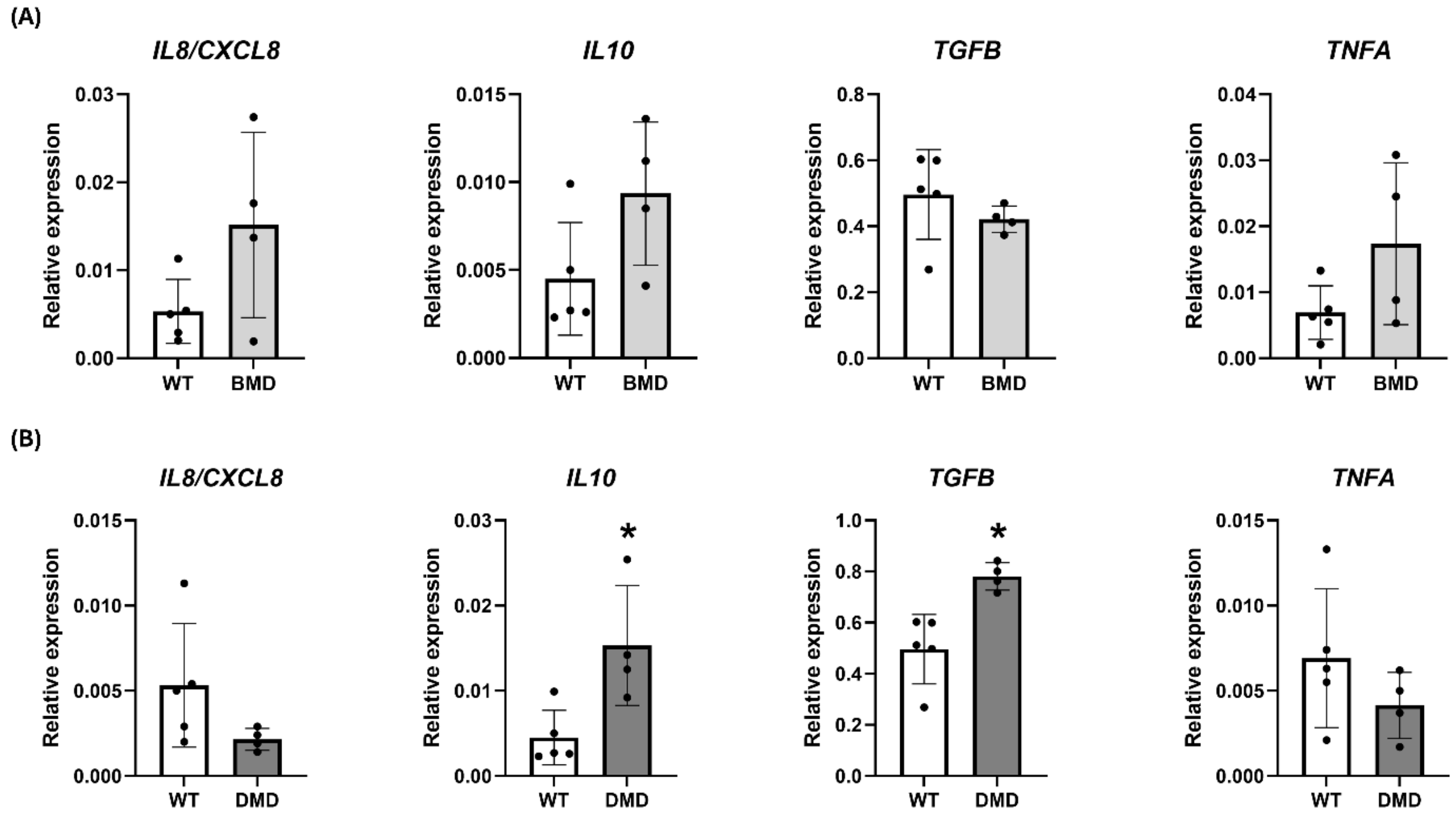

Fig. S3 Independent validation of RNA-seq data by qRT-PCR. Relative mRNA expression of selected inflammatory genes (*IL8*, *IL10*, *TGFB*, and *TNFA*) was quantified by qRT-PCR in an independent set of triceps brachii muscle samples from wild-type (WT), *DMD*Δ52 (DMD), and *DMD*Δ51–52 (BMD) pigs (n = 4–5 per group). Expression levels were normalized to the housekeeping gene *GAPDH*. Statistical significance was assessed by a t-test (*$p < 0.05$). The qRT-PCR results closely corroborate the differential expression patterns observed in the RNA-seq analysis.

(A)

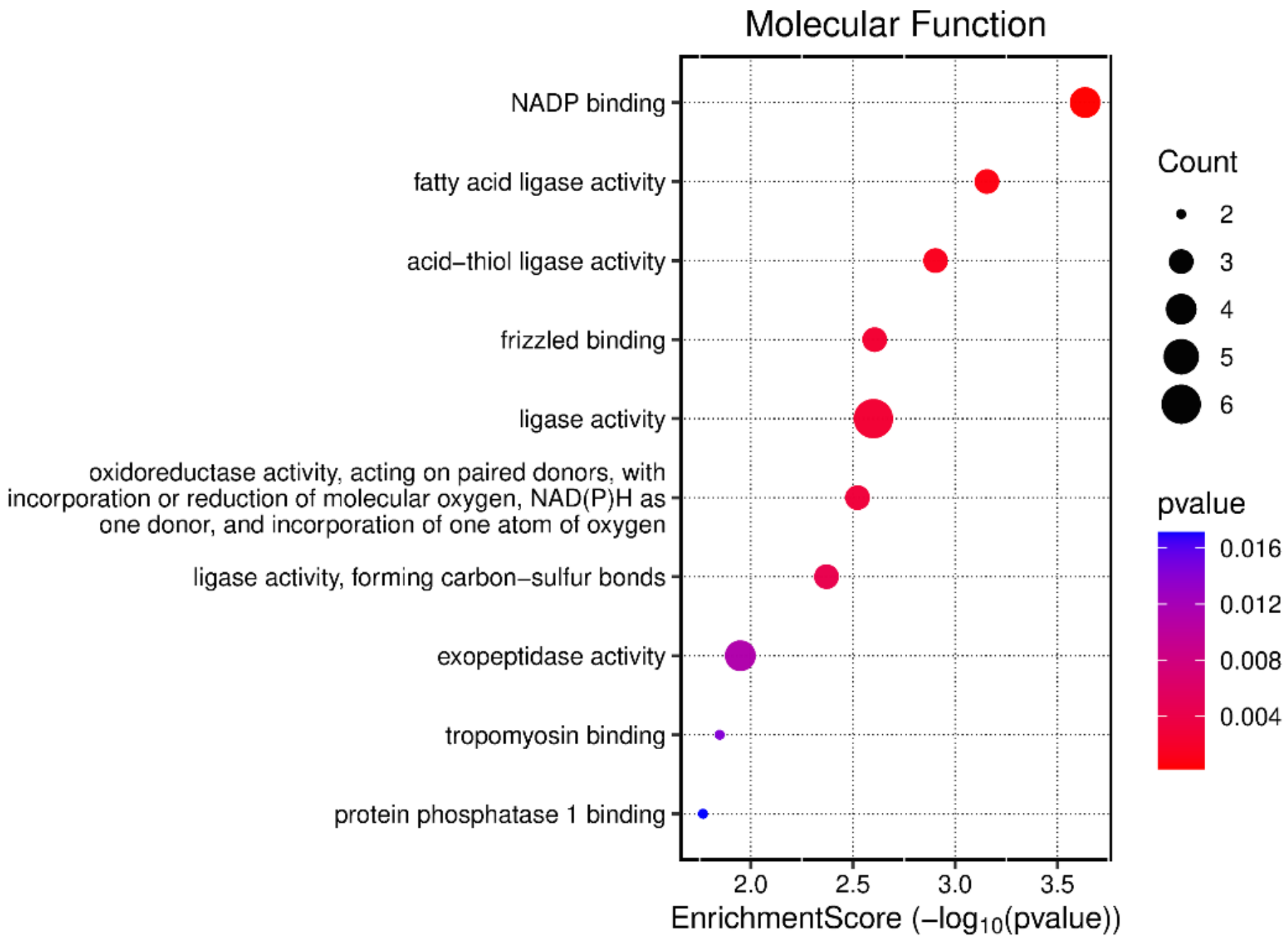

(B)

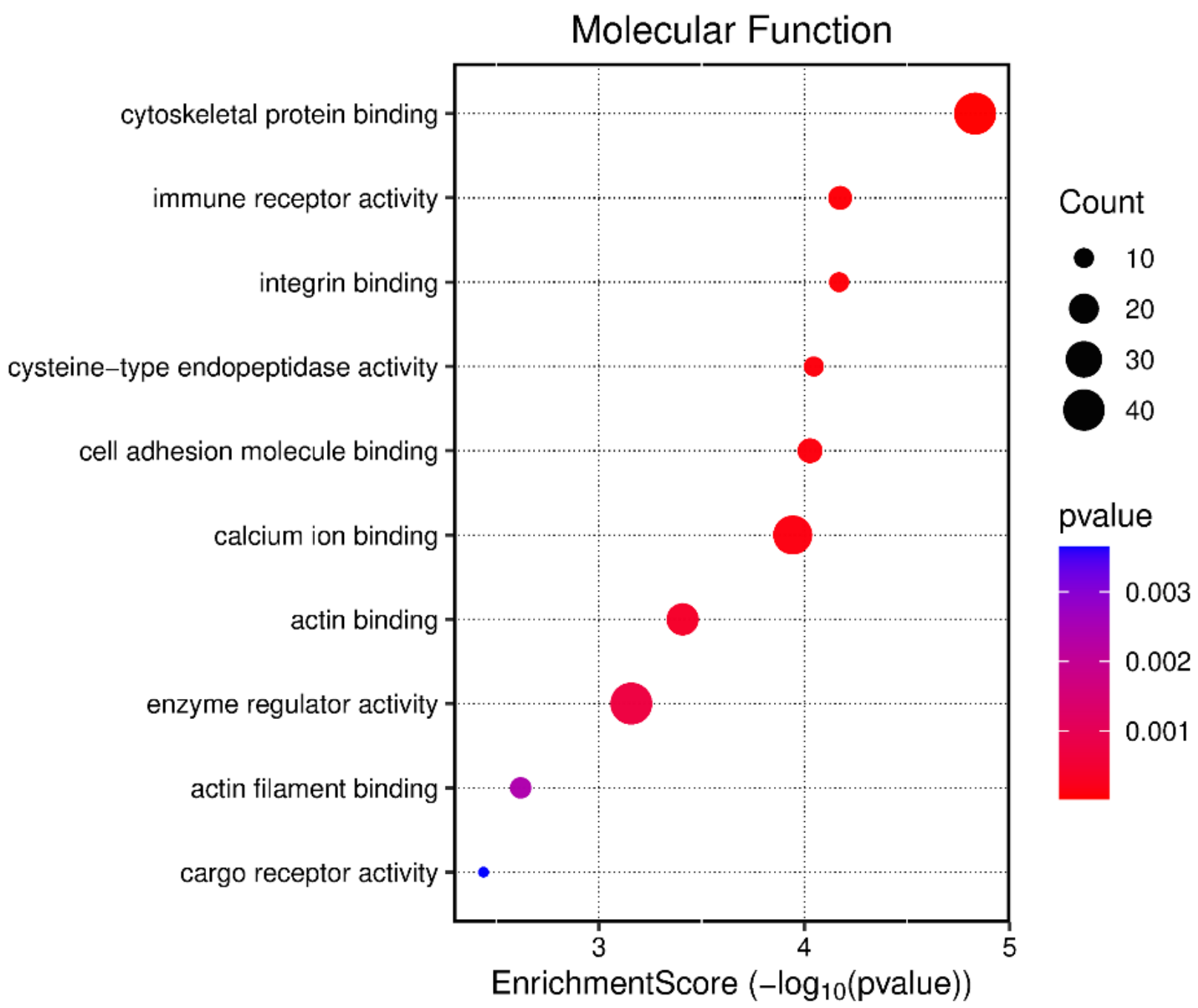

Fig. S4 (A) Dot plot displaying the top 10 enriched molecular functions by the genes uniquely downregulated in DMD skeletal muscle. (B) Dot plot displaying the top 10 enriched molecular functions by the genes uniquely upregulated in DMD skeletal muscle. Dot size reflects the number of genes per pathway, and color indicates statistical significance.

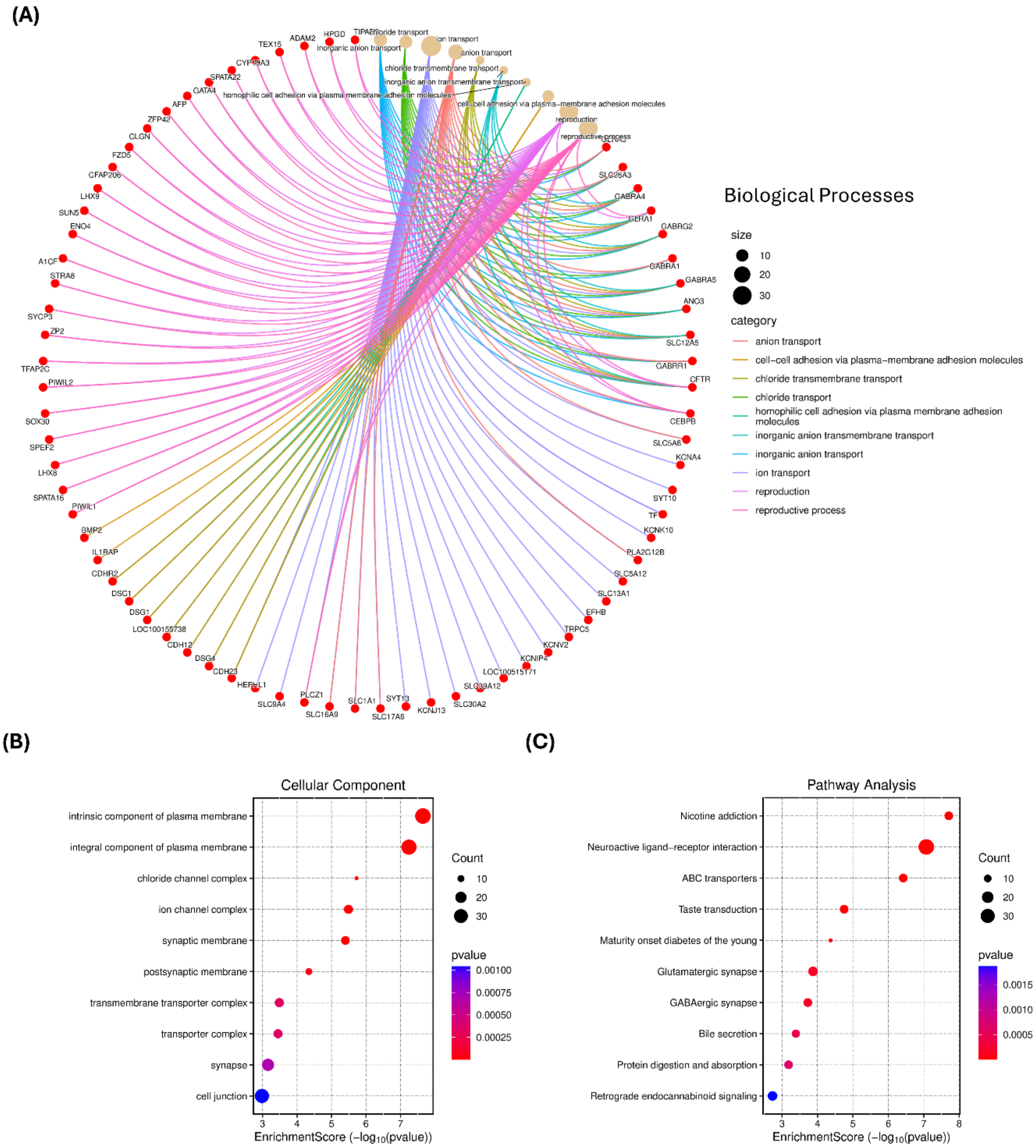

Fig. S5 Enrichment analysis of genes uniquely upregulated in BMD muscle. (A) CNET plot showing the interconnected network of enriched BPs and their associated genes. Pathway nodes indicate functional categories, while gene nodes highlight shared contributions across multiple pathways. (B) Dot plot displaying the top 10 enriched CCs and (C) KEGG pathways. Dot size reflects the number of genes per pathway, and color indicates statistical significance.

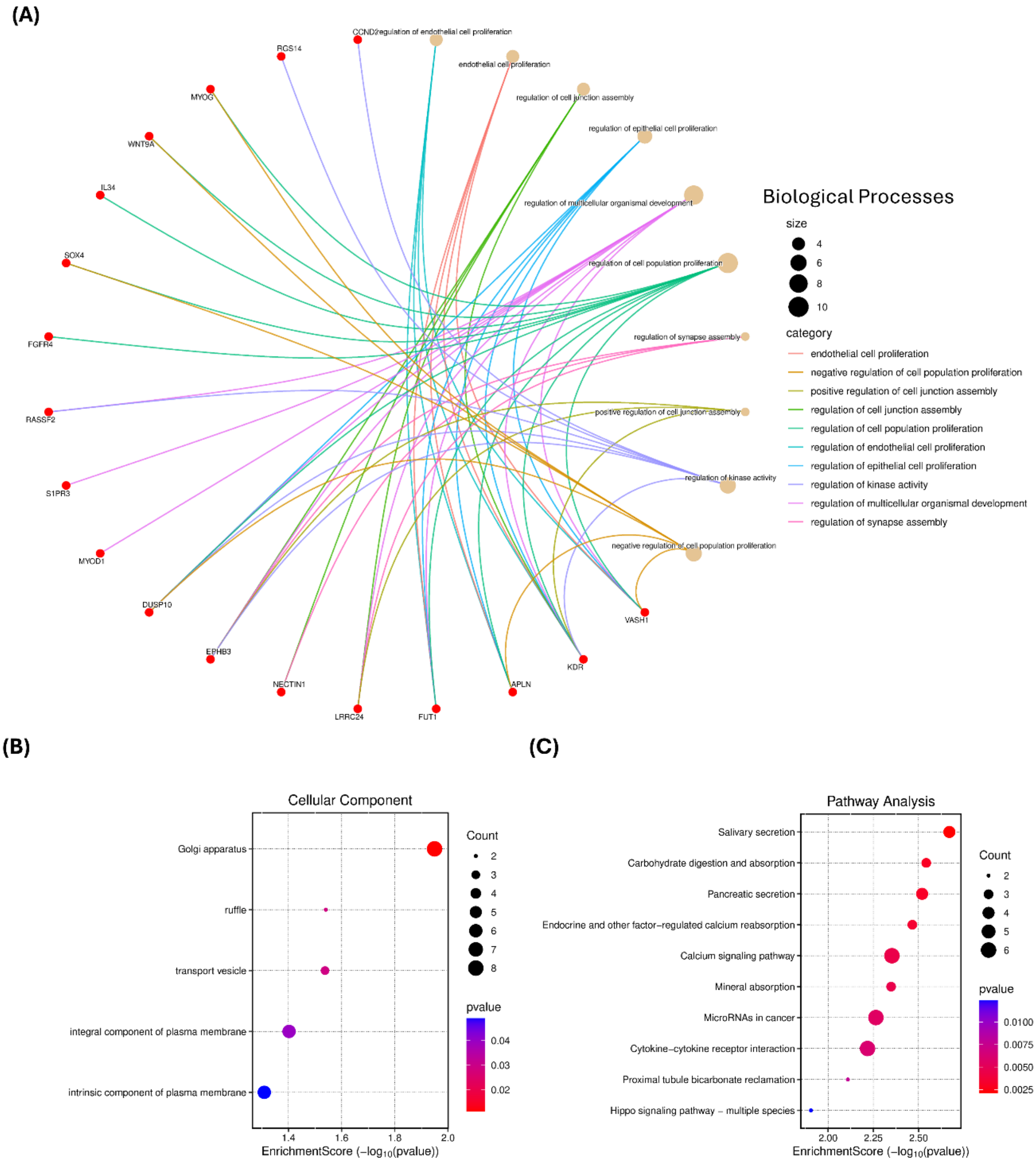

Fig. S6 Enrichment analysis of genes uniquely downregulated in BMD muscle. (A) CNET plot showing the interconnected network of enriched BPs and their associated genes. Pathway nodes indicate functional categories, while gene nodes highlight shared contributions across multiple pathways. (B) Dot plot displaying the top 10 enriched CCs and (C) KEGG pathways. Dot size reflects the number of genes per pathway, and color indicates statistical significance.

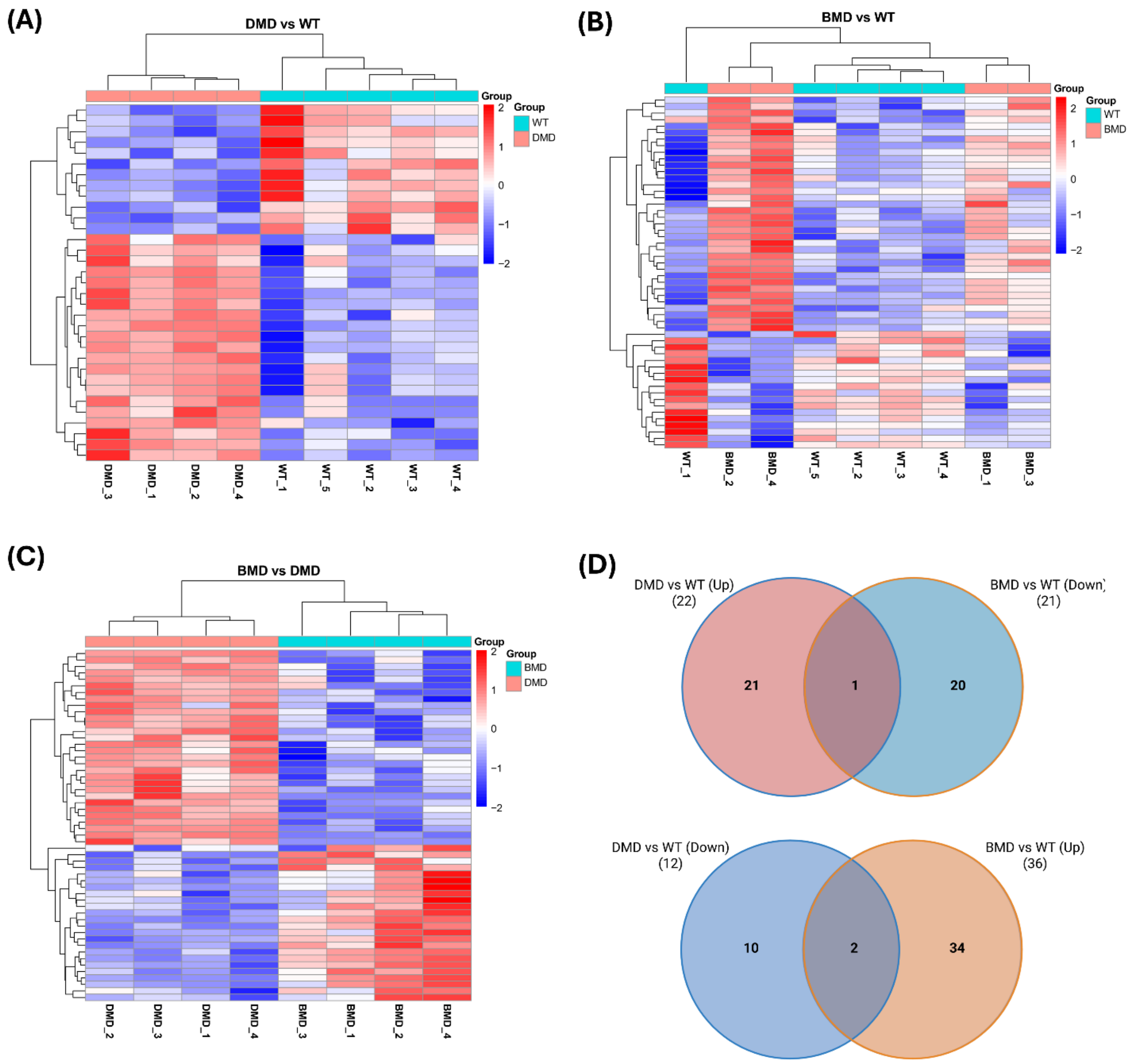

Fig. S7 Differential miRNA expression analysis in skeletal muscle from DMD, BMD, and WT pigs. Heatmaps of differentially expressed miRNAs in pairwise comparisons: (A) DMD versus WT, (B) BMD versus WT, and (C) BMD versus DMD. Each column represents a biological replicate (n=4-5 per group), and each row corresponds to a differentially expressed miRNA (|log2FC| ≥ 1, adjusted p-value ≤ 0.05). Expression values were z-transformed, with blue indicating lower expression and red indicating higher expression. The color bar at the top denotes the experimental groups: WT (green), BMD (red), and DMD (blue). Hierarchical clustering highlights distinct expression patterns across genotypes. (D) Comparison of upregulated miRNAs in DMD versus WT, with downregulated miRNAs in BMD versus WT, and the comparison of downregulated miRNAs in DMD versus WT, with upregulated miRNAs in BMD versus WT.

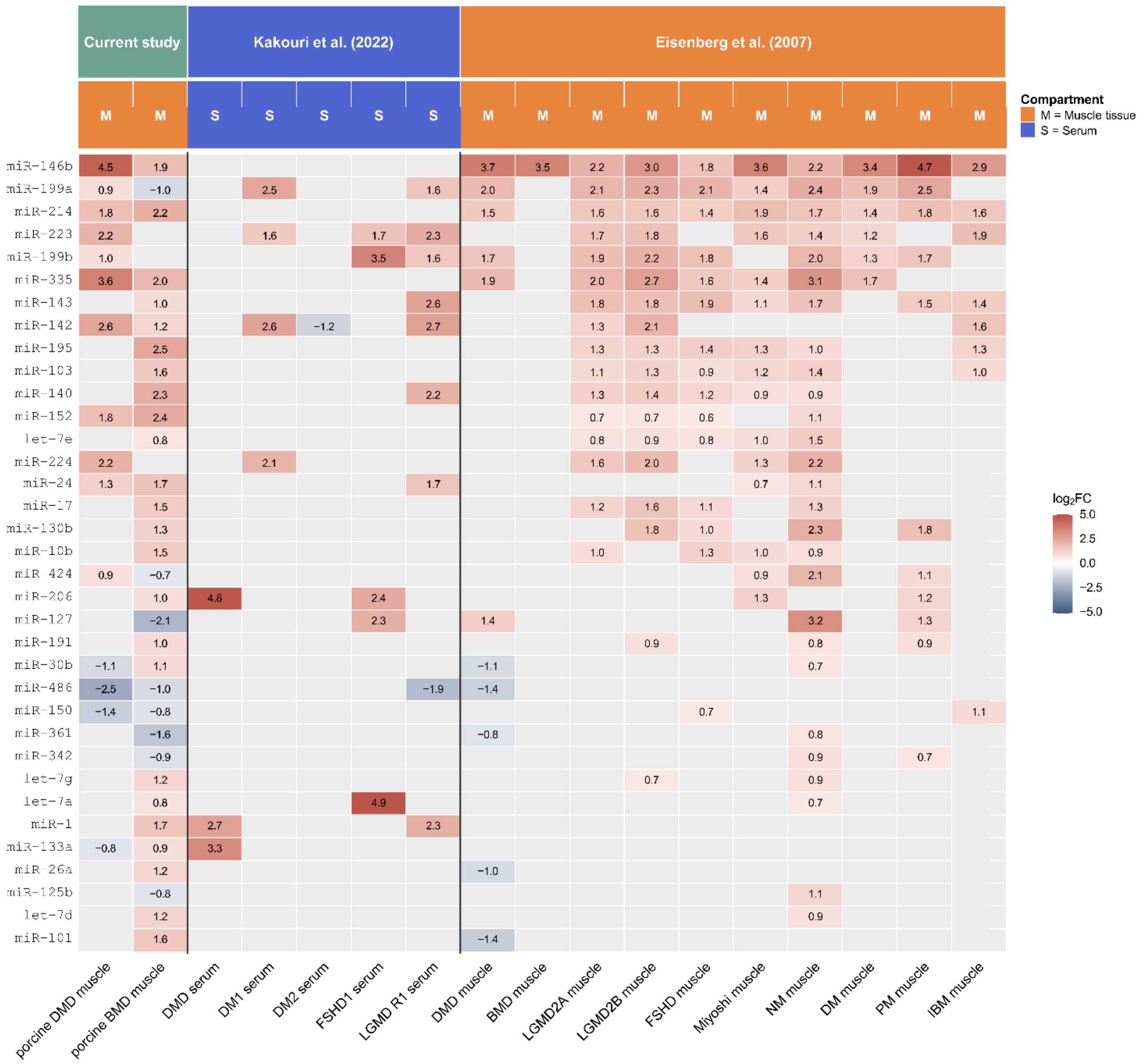

Disease abbreviations:
DMD = Duchenne muscular dystrophy; BMD = Becker muscular dystrophy.
DM1 = Myotonic dystrophy type 1; DM2 = Myotonic dystrophy type 2; DM = Dermatomyositis (Eisenberg cohort only; distinct from DM1/DM2)
FSHD = Facioscapulohumeral muscular dystrophy; FSHD1 = Facioscapulohumeral muscular dystrophy type 1.
LGMD R1 = Limb−girdle MD recessive 1 (calpainopathy, former LGMD2A).
LGMD2A = Limb−girdle MD 2A (calpainopathy); LGMD2B = Limb−girdle MD 2B (dysferlinopathy).
Miyoshi = Miyoshi distal myopathy (dysferlinopathy); NM = Nemaline myopathy; PM = Polymyositis; IBM = Inclusion body myositis.

Grey cells: the miRNA was not significantly differentially expressed in the respective disease context.

Fig. S8 Supplementary Figure S7: Cross-species and cross-studies comparison of differentially expressed miRNAs in the current study. Heatmap of log2 fold changes for 35 miRNAs (rows) detected as significantly differentially expressed (DE) in at least one of the samples analyzed (columns) by Kakouri et al. (2022) or Eisenberg et al. (2007). The analysis spnas three independent studies: the current porcine skeletal muscle dataset (current study; two contexts: porcine DMD, porcine BMD), the human serum miRNA profile of Kakouri et al. (five neuromuscular diseases: DMD, DM1, DM2, FSHD1, LGMD-R1; top-20 DE miRNAs per disease), and the human muscle biopsy miRNA microarray dataset of Eisenberg et al. (ten neuromuscular disorders: DMD, BMD, LGMD2A, LGMD2B, FSHD, Miyoshi, NM, DM, PM, IBM). The upper annotation strip indicates the study origin (Current study / Kakouri et al. / Eisenberg et al.); the lower annotation strip distinguishes the analyte pool (M = muscle tissue, S = serum). Tile color encodes log2FC clipped to [−5, +5] (blue = downregulated, white = 0, red = upregulated) to keep individual outliers from dominating the diverging scale; the number inside each tile shows the unclipped log2FC. Rows are ordered by number of contexts with a DE signal (most frequently replicated miRNAs at the top). Disease abbreviations are expanded beneath the heatmap.

miRNA identifiers were harmonized to a species-agnostic core name by removing the ssc-/has- prefix. For strand resolution, the 3p/5p suffix was retained where annotated; because the 2007 Eisenberg microarray platform did not distinguish strands, its entries were matched at the suffix-stripped name level. Eisenberg's signed linear fold changes were converted to log2 fold changes according to log2FC = sign(fold) × log2(|fold|). No new statistical test was applied across studies. Every entry in the replication map represents a significant DE call by the threshold of the original study. This comparison identified 35 miRNAs that we reproducibly dysregulated across species and analyte pools

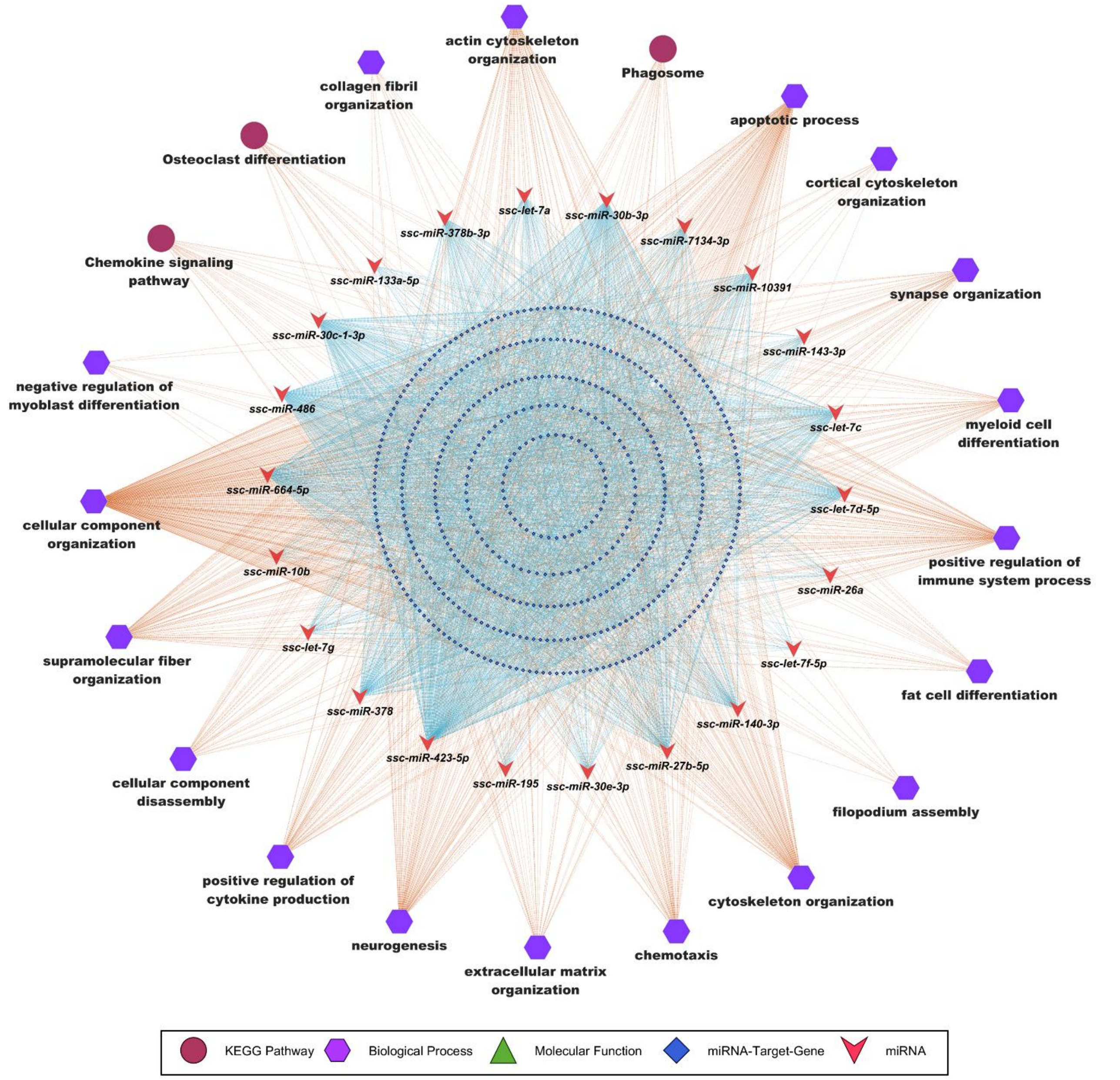

Fig. S9 Functional enrichment analysis of upregulated miRNAs in BMD relative to DMD and their downregulated target genes. A total of 86 downregulated DEGs, identified as targets of 30 upregulated miRNAs, were subjected to enrichment analysis. The figure illustrates representative enriched terms and pathways, along with the associated miRNAs. Terms and pathways shown passed the significance threshold of $p \leq 0.05$. A complete list of significantly enriched terms and pathways associated with this gene set is provided in Supplementary Table 6.
